# Mitochondrial Genomes Reveal Global Population Structure, Lineage Divergence, and Candidate Diagnostic Markers of *Diaphorina citri*

**DOI:** 10.3390/insects17090917

**Published:** 2026-09-01

**Authors:** Lijuan Yin, Suming Dai, Dan Wei, Dazhi Li, Yunsheng Wang

**Affiliations:** 1 Hunan Provincial Key Laboratory for Biology and Control of Plant Diseases and Insect Pests, College of Plant Protection, Hunan Agricultural University, Changsha 410128, China; yinlijuan1005@163.com; 2 Yuelushan Laboratory, Changsha 410128, China; dsm531@126.com; 3 College of Horticulture, Hunan Agricultural University, Changsha 410128, China; 17674071649@163.com

**Keywords:** Asian citrus psyllid, genetic differentiation, huanglongbing, mitochondrial genome, phylogeography, single nucleotide polymorphism

## Abstract

The Asian citrus psyllid, *Diaphorina citri* Kuwayama (Hemiptera: Liviidae), is the primary vector of *Candidatus Liberibacter asiaticus*, the causal agent of huanglongbing, the most devastating disease of citrus worldwide. Understanding its population structure and developing lineage-specific markers are important for surveillance, quarantine and pest management. Here, 133 complete mitochondrial genomes were analyzed, comprising 77 previously published sequences, 13 newly assembled genomes and 43 metagenome-assembled mitogenomes. Phylogenetic analysis revealed two highly differentiated mitochondrial lineages—an Asian lineage (*n* = 91, including the Uruguay sample) and an American lineage (*n* = 42, including the Pakistan sample) (FST = 0.560, *p* < 0.001)—separated by 32 fixed variants (132 genomes, excluding the reference sequence MF426268.1). Within the Asian lineage, the Yunnan–Guizhou Plateau population showed significant differentiation from lowland populations (FST = 0.130, *p* < 0.05), reduced nucleotide diversity and moderately supported monophyletic clustering consistent with long-term isolation, while demographic analyses suggested that Southeast China may have acted as a post-colonization expansion center. Candidate lineage-specific markers, including variants in *trnG* and *rrn16*, provide molecular resources for population-origin assignment, quarantine surveillance and pest monitoring; these candidates require validation across broader geographic sampling.

## Simple Summary

The Asian citrus psyllid, *Diaphorina citri*, transmits the bacterium that causes citrus huanglongbing, the most destructive citrus disease worldwide, and tracing where psyllid populations come from is essential for quarantine and surveillance. In this study, 133 complete mitochondrial genomes from Asia, America, and Pakistan were assembled and compared. The genomes fall into two deeply diverged lineages (Asian and American) separated by 32 fixed nucleotide differences (132 genomes, excluding the reference sequence MF426268.1), and the Yunnan–Guizhou Plateau population forms a genetically distinct subgroup carrying unique variants in the *trnG* and *rrn16* genes. These lineage-specific mutations are candidate molecular markers that could help determine the geographic origin of psyllids intercepted at ports of entry.

## 1. Introduction

The Asian citrus psyllid, *Diaphorina citri* Kuwayama (Hemiptera: Liviidae), is recognized as one of the most economically important pests of citrus worldwide [1]. As the primary vector of *Candidatus Liberibacter asiaticus*, the bacterial pathogen responsible for Huanglongbing (HLB), also known as citrus greening disease, *D. citri* poses a severe threat to global citrus production [2,3]. HLB has caused devastating losses in citrus-growing regions across Asia, the Americas, and Africa, with no effective cure currently available [4,5]. The disease has resulted in billions of dollars in economic losses and has fundamentally altered citrus production practices worldwide [6]. The global distribution of *D. citri* reflects both natural dispersal and human-mediated transport through the international trade of citrus plants and plant materials [7,8]. Originally endemic to Asia, the pest has established populations in the Americas (first detected in Florida in 1998 and Brazil in the 1940s) [9,10], Africa [11], and various island territories [12]. Understanding the population structure, genetic diversity, and dispersal pathways of *D. citri* is essential for developing effective pest management strategies, tracking invasion routes, and predicting future spread patterns [13].

Previous mitochondrial studies using the cytochrome oxidase I (*COI*) gene have revealed genetic differentiation between Asian and American populations of *D. citri* and have been widely used to characterize population structure across South America, North America, India, Iran and Pakistan [14,15,16]. Based on *COI* sequences, studies have proposed a southern/southeastern Asian origin and three trans-oceanic expansion routes for *D. citri*, and phylogenetic analyses have suggested recent dispersal from Southeast Asia to China and across the seas to Africa and the Americas [14]. However, these studies were limited by single-gene markers and relatively small sample sizes, providing incomplete insight into the complex population structure and evolutionary history of this species.

Single-locus analyses have inherent limitations; multi-locus and whole-genome comparisons are increasingly recognized as improving the accuracy of population genetic inference. Based on mitochondrial *COI* and cytochrome b (*Cytb*) sequences, two putative invasion sources (Guangdong and Yunnan) and three subsequent dispersal pathways have been proposed for *D. citri* in China: expansion from Yunnan across the Yunnan–Guizhou Plateau; spread from Guangdong to Guangxi and Guizhou; and eastward movement from Guangdong to Fujian, with a minor extension into Jiangxi [17]. Higher-resolution genomic data are needed to test these inferred routes and clarify the timing and directionality of dispersal events [18].

Mitochondrial genomes are widely used in phylogeographic and population genetic studies due to their maternal inheritance, lack of recombination, relatively fast evolutionary rate, and high copy number [19]. Compared to single-gene markers, complete mitogenomes provide substantially greater phylogenetic resolution, enabling more accurate inference of evolutionary history and the identification of population-specific variants that can be developed as candidate diagnostic markers for tracing pest origins and movement pathways [20,21]. The complete mitochondrial genome of *D. citri* consists of 13 protein-coding genes, 2 rRNA genes, 22 tRNA genes, and a variable control region. This genomic architecture has enabled high-resolution population delineation. Analyses of whole mitochondrial genomes from China, the USA, and Pakistan defined three major genetic groups: MG1 in Southwest China, MG2 in Southeast China and Southeast Asia, and MG3 in the Americas and West Asia. More recent mitogenomic surveys have further resolved Chinese populations into three distinct clades: Clade A in Central-East China, Clade B along the Jinsha River in Sichuan, and Clade C in Southwest China, encompassing Yunnan, Sichuan, and Guizhou [22]. However, these mitogenome-based classifications are based on limited sampling, and robust phylogeographic inference requires a larger number of geographically representative mitochondrial genomes to validate existing groupings and uncover finer-scale population structure.

China, as part of the native range of *D. citri*, harbors substantial genetic diversity of this pest species [23]. Within China, diverse topographical features, including the Yunnan–Guizhou Plateau, may act as geographical barriers influencing population structure and promoting local adaptation [24]. The Yunnan–Guizhou Plateau region is characterized by high elevation (average >1000 m), unique climatic conditions, and complex terrain, which may impose selective pressures on *D. citri* populations and lead to genetic differentiation from lowland populations [25].

In this study, 133 complete mitochondrial genomes of *D. citri* were assembled and analyzed, representing the largest dataset to date, including samples from Asia (China, including Taiwan Province, Southeast Asian countries and Pakistan), the Americas (USA and Uruguay), and Africa (Kenya and Réunion). The objectives were to (1) characterize the global population genetic structure and phylogenetic relationships of *D. citri*; (2) identify fixed nucleotide differences that could serve as candidate lineage-specific molecular markers; (3) examine genetic differentiation between highland (Yunnan–Guizhou Plateau) and lowland populations within the Asian mitochondrial lineage; and (4) evaluate demographic expansion and historical connectivity using population genetic analyses. The findings enhance understanding of *D. citri* population history and provide candidate molecular resources for pest monitoring, quarantine and integrated pest management.

## 2. Materials and Methods

### 2.1. Mitochondrial Genome Data Collection

Complete mitochondrial genome sequences of *D. citri* were obtained from three sources. First, 77 previously published mitochondrial genomes were downloaded from the National Center for Biotechnology Information (NCBI) GenBank database, including reference sequences and whole-genome sequencing data [26]. Second, 13 mitochondrial genomes were newly assembled from samples collected in Hunan Province, China, using short-read sequencing data (see below) [27]. Third, 43 gut metagenomic sequencing datasets of *D. citri* were downloaded from China and the USA ([28]; accession numbers: SRR16895067–SRR16895113; SRR23454940, SRR23454942, SRR23454943; SRR16559822, SRR16559823).

The 13 Hunan samples were collected in 2025 from 13 counties across Hunan Province, China (Shimen, Luxi, Lianyuan, Huayuan, Dongkou, Yizhang, Chenxi, Baojing, Liuyang, Lengshuitan, Anxiang, Shuangfeng and Dingcheng). For each sample, 5–10 adult psyllids were flash-frozen in liquid nitrogen, homogenized using a Tissuelyser-48 grinder (Shanghai Jingxin, Shanghai, China) with steel beads, and genomic DNA was extracted using a Tiangen genomic DNA extraction kit (Tiangen Biotech, Beijing, China) following the manufacturer’s instructions. Sequencing libraries of 300–400 bp were constructed on the DNBSEQ-T7 platform (MGI, Shenzhen, China) and sequenced on an MGISEQ-2000 instrument (MGI, Shenzhen, China), yielding an average of 23.14 Gb of data per sample (mean sequencing depth 49.66×). Mitochondrial genomes were assembled with GetOrganelle v1.7.7.1, annotated with GeSeq (https://chlorobox.mpimp-golm.mpg.de/geseq.html, accessed on 27 April 2025), and variants were called with GATK v4.6.1.0 (Broad Institute, Cambridge, MA, USA); consensus sequences were generated as described in [27]. The raw sequencing data were deposited in the China National GeneBank DataBase (CNGBdb) under BioProject PRJCA055168 [27].

The final dataset comprised 133 mitochondrial genomes representing samples from China (*n* = 75, including Taiwan Province), USA (*n* = 41), Kenya (*n* = 2), Réunion Island (*n* = 9), Uruguay (*n* = 1), Pakistan (*n* = 1), Cambodia (*n* = 1), Malaysia (*n* = 1), Vietnam (*n* = 1), and Indonesia (*n* = 1). Detailed sample information is provided in Table S1.

All mitochondrial genome sequences were quality-checked, and basic genomic statistics including total length and GC content were calculated for each sample. For samples collected from Sichuan, Yunnan, and Guizhou provinces (*n* = 8), sampling locations were mapped against 90 m resolution Digital Elevation Model data obtained from the Geospatial Data Cloud platform (http://www.gscloud.cn, accessed on 10 December 2025) using ArcGIS 10.8.2 (Esri, Redlands, CA, USA). The average elevation of these eight sampling sites was 1166 m (Table S2), a substantial elevational contrast with the lowland populations in Hunan, Guangdong, and Jiangxi provinces. In addition, one accession previously listed as NC_036352.1 in Table S1 was corrected to SRR23454944 (a sample from Guangxi, China); the sequence bearing the true NC_036352.1 accession was never included in any analysis of this study.

### 2.2. Mitochondrial Genome Assembly

Paired-end whole-genome sequencing (WGS) data for 43 *D. citri* samples (38 from the USA and 5 from China) were retrieved from the NCBI Sequence Read Archive (SRA) using the SRA Toolkit (v3.0.0) fasterq-dump command. The 13 Hunan mitogenomes were newly sequenced and assembled in this study with a dedicated organelle-genome assembler (Section 2.1); all mitogenomes from the three sources were subsequently processed through the same alignment, polymorphism-detection and phylogenetic analyses.

Metagenomic assembly was performed using MEGAHIT v1.2.9 with paired-end reads as input. The following parameters were used: minimum contig length of 500 bp, k-mer sizes ranging from 21 to 141 bp with a step size of 20 (--k-min 21 --k-max 141 --k-step 20), 80% of available memory (--memory 0.8), and 8 CPU threads.

Mitochondrial contigs were extracted from the metagenomic assemblies by mapping all assembled contigs against the reference *D. citri* mitochondrial genome (NCBI accession MG489916.1, 15,020 bp) using minimap2 v2.24 with the asm5 preset. Only contigs that successfully aligned to the reference were retained as candidate mitochondrial sequences using Samtools v1.16 and seqtk subseq (https://github.com/lh3/seqtk, accessed on 20 May 2025). For samples yielding more than one mitochondrial contig, a second round of assembly was performed using MEGAHIT with 3 million randomly subsampled read pairs (seed = 200), followed by repeated mitochondrial contig extraction.

Quality assessment of metagenome-assembled mitochondrial genomes. Assembly quality was systematically evaluated for all 43 samples by aligning each assembled mitogenome to the reference (MG489916.1) using minimap2. All 43 assemblies (100%) were recovered as a single contig. The mean assembly length was 15,071 ± 105 bp (range: 14,609–15,356 bp), corresponding to 100.5 ± 0.7% of the reference length. Mean reference sequence identity was 99.50 ± 0.16% (median: 99.45%; range: 99.24–99.93%), with all 43 assemblies (100%) achieving ≥99% identity. Mean reference coverage was 99.80 ± 0.73% (median: 100.0%; range: 95.26–100.0%), with 42 assemblies (97.7%) achieving ≥99% coverage and all 43 (100%) achieving ≥95% coverage. The mean GC content was 25.44 ± 0.12%, consistent with the reference genome (25.53%). The median number of mismatches per assembly was 83 (IQR: 80–84), and the median number of gaps was 8. All assemblies were placed within their expected geographic clades in the haplotype network and maximum-likelihood tree, with no anomalous placement suggestive of cross-contamination. The complete quality assessment results are provided in Table S3.

### 2.3. Circularization and Gap Filling

To reconstruct complete circular mitochondrial genomes, the extracted mitochondrial contigs were processed through a circularization pipeline. First, for samples with multiple contigs, reference-guided scaffolding was performed using RagTag v2.1.0 with minimap2 as the aligner. If RagTag failed, ABACAS v1.3.1 was used as an alternative scaffolding tool.

Gaps within scaffolded sequences were filled using SOAPdenovo2-GapCloser v1.12 (https://github.com/aquaskyline/SOAPdenovo2, accessed on 22 May 2025) with the original paired-end reads as input. The resulting gap-filled sequences were then circularized using Circlator v1.5.5, which uses the original sequencing reads to identify and resolve the circularization point. If Circlator failed with default parameters, a second attempt was made with adjusted parameters (--b2r_length_cutoff 500 --assemble_spades_k 21, 33, 55, 77). For samples where Circlator could not produce a circularized sequence, the gap-filled scaffold was retained as the final assembly.

### 2.4. Phylogenetic Tree Construction and Polymorphic Site Analysis

**Figure 1:**
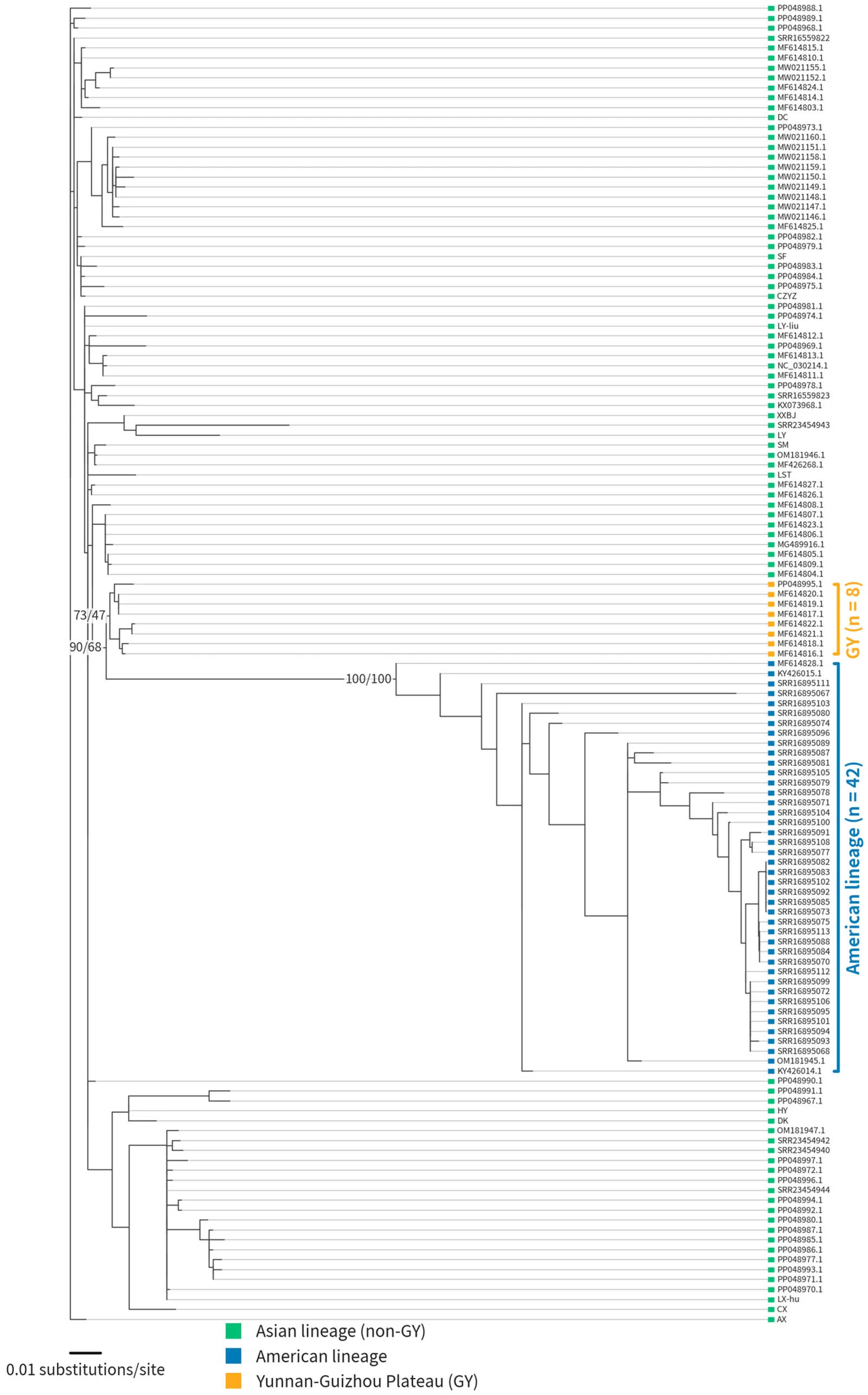
Partitioned maximum-likelihood phylogenetic tree of 133 *Diaphorina citri* mitochondrial genomes inferred with IQ-TREE v3.1.3 (MFP + MERGE; two final partitions; see Section 2.4). Tip label colors indicate mitochondrial lineage and regional group: green = Asian lineage (including Uruguay), blue = American lineage (*n* = 42, including Pakistan), and orange = Yunnan–Guizhou Plateau population (GY, *n* = 8). SH-aLRT/ultrafast bootstrap support values (%, 1000 replicates each) are shown at major nodes. The American lineage was strongly supported (100/100), whereas the GY clade received moderate support (73.1/47). The scale bar represents nucleotide substitutions per site.

Multiple sequence alignment of the 133 mitochondrial genomes was performed using MAFFT v7.520 with default parameters [29]. The alignment was based on full-length mitochondrial DNA sequences to ensure homology across all sites. Maximum-likelihood phylogenetic trees were inferred using IQ-TREE v3.1.3 [30]. Two analyses were conducted: an unpartitioned analysis and a partitioned analysis in which the alignment was divided into 14 initial partitions (13 protein-coding genes and one non-coding partition) with partition merging enabled (-m MFP + MERGE). The best-fitting substitution models were selected automatically using ModelFinder [31] under the Bayesian information criterion, yielding TIM2 + F + R4 for the unpartitioned analysis (log-likelihood −27,719.972) and two merged partitions in the partitioned analysis (protein-coding genes: TN + F + I; non-coding regions: TIM2 + F + I + G4; log-likelihood −27,044.417). Node support was assessed with 1000 SH-aLRT replicates and 1000 ultrafast bootstrap replicates (-alrt 1000 -B 1000 -bnni). The partitioned tree, which recovered the same major clades as the unpartitioned tree, was used for presentation (Figure 1). The resulting tree was uploaded to the Interactive Tree of Life (iTOL, https://itol.embl.de/, accessed on 22 August 2026) for visualization and annotation.

Nucleotide polymorphism and genetic variation sites across the 133 samples were detected using DnaSP v6.12 software [32] based on the MAFFT-aligned mitochondrial genome sequences. This analysis identified all polymorphic sites and calculated nucleotide diversity metrics for the entire dataset.

### 2.5. Mitochondrial Genome-Based Haplotype Network Construction

Haplotype identification and lineage relationship inference were performed using DnaSP v6.12 [32] and PopART v1.7 [33] based on the 133 complete mitochondrial genome sequences. DnaSP was used to define haplotypes from nucleotide variation sites, with identical sequences assigned to the same haplotype; the number of haplotypes (H), haplotype frequencies, geographic distribution of each haplotype, and haplotype diversity (Hd) with its variance and standard deviation were calculated.

A median-joining (MJ) network was then constructed in PopART v1.7 under default parameters, with median vectors enabled to infer unobserved intermediate ancestral haplotypes.

### 2.6. Global Population Genetic Structure and Dispersal Pathway Inference

Due to severe sampling imbalance across regions, quantitative population genetic analyses (pairwise comparisons, TreeMix, and AMOVA) were restricted to populations with *n* ≥ 4: Southeast Asia (SA, *n* = 4, Cambodia, Malaysia, Vietnam, Indonesia), Southeast China (SC, *n* = 67), Yunnan–Guizhou Plateau (GY, *n* = 8), North America (NA, *n* = 41), and Africa (AF, *n* = 11). Populations represented by single individuals (Pakistan, Uruguay) were excluded from quantitative geographic-population analyses but retained for qualitative phylogenetic placement and haplotype-network construction. The small SA sample size (*n* = 4) is acknowledged as a limitation that may affect the reliability of gene-flow directionality inference. For lineage-level comparisons, samples were assigned to the Asian mitochondrial lineage (*n* = 91, including Uruguay) or the American mitochondrial lineage (*n* = 42, including Pakistan) according to phylogenetic placement. These lineage labels describe mitochondrial ancestry rather than sampling geography.

To compare geographic populations, pairwise population genetic analyses were conducted using DnaSP v6.12 [32] on the 131 samples assigned to the five retained populations. Populations were defined as follows: Southeast Asia (SA, *n* = 4, including Cambodia, Vietnam, Indonesia, and Malaysia); Southeast China (SC, *n* = 67, including Hunan, Guangdong, Fujian, Zhejiang, Hainan, Guangxi, Taiwan Province and Jiangxi); Yunnan–Guizhou Plateau (GY, *n* = 8, including samples from Yunnan, Guizhou, and Sichuan at high elevation); Africa (AF, *n* = 11, including Kenya and Réunion); and North America (NA, *n* = 41, USA). Pakistan and Uruguay were not assigned to quantitative geographic populations because each was represented by a single sample. The assignment of samples to GY versus SC was based on genetic clustering in the haplotype network rather than on administrative boundaries or elevation alone: two samples collected on the Yunnan–Guizhou Plateau (Changshun, Guizhou, PP048996.1, 676 m; Leibo, Sichuan, PP048997.1, 1360 m) carried plains-type haplotypes and were therefore assigned to SC. A sensitivity analysis using a geography-based assignment is reported in Section 3.3.

For each pair of populations, the following parameters were calculated: (1) nucleotide diversity estimated separately for each of the two populations to assess average nucleotide diversity; (2) Watterson’s estimator to evaluate historical effective population size and mutation rate; (3) number of shared mutations between populations; (4) ratio of private mutations (unique polymorphisms in each population), calculated as A:B, to infer directionality of expansion; (5) fixed differences, defined as single nucleotide variants present in one population but completely absent in the other; and (6) net genetic differentiation (Da), reflecting net genetic distance between populations, but Da is not used here to infer dispersal directionality or gene-flow intensity.

Demographic scenario comparison was performed using DIYABC v2.0 [34,35] to infer the most likely divergence history among the Southeast Asia (SA), Southeast China (SC), and Yunnan–Guizhou Plateau (GY) populations. Three competing divergence scenarios were compared (Table S8): Scenario 1 (SA → SC → GY, sequential divergence with Southeast Asia as the ancestral source), Scenario 2 (Southeast China as the ancestral source), and Scenario 3 (GY as the earliest-diverging lineage, with SC and SA forming a sister group). Each scenario was simulated 1,000,000 times under uniform prior distributions for effective population sizes (N1, GY: 1 × 10^5^–1 × 10^7^; N2, SC: 1 × 10^3^–1 × 10^5^; N3, SA: 5 × 10^4^–8 × 10^5^) and divergence times (t1, GY–SC divergence: 1 × 10^3^–5 × 10^4^ generations; t2, SC–SA divergence: 1 × 10^4^–1 × 10^5^ generations; Table S9). Summary statistics included one-sample statistics (mean pairwise differences, number of segregating sites, Tajima’s D) and two-sample statistics (FST, shared and private polymorphisms). Model choice was performed using both the direct approach (based on the 500 simulated datasets closest to the observed data) and logistic regression (based on the 10,000 closest datasets, using linear discriminant analysis components); a scenario was considered supported only when the two procedures concordantly selected it.

Additionally, maximum-likelihood tree-based migration analysis was conducted using TreeMix (v1.13) [36] to model population splits and admixture events among the five geographic populations (SA, SC, GY, NA, AF). A total of 1219 biallelic loci (complete data across the 131 samples assigned to the five retained populations) were used as input. Population allele frequencies were computed for each locus, and a block size of 500 loci (-k 500) was specified to account for linkage disequilibrium. Maximum-likelihood trees were constructed for migration parameters m = 0 to 4 with global rearrangements enabled (-global) and a fixed random seed (-seed 20260822) to ensure reproducibility, and models were compared by log-likelihood. Bootstrap validation was performed with 100 replicates (resampling loci with replacement) under m = 0 to assess topology stability, each replicate using -global and a replicate-specific seed; under m = 1, convergence was evaluated using ten time-limited (1 h) test replicates (Figure S3).

Analysis of Molecular Variance (AMOVA) was conducted to evaluate hierarchical genetic structure among groups. Population differentiation was quantified using FST statistics, with significance determined by 10,000 permutation tests in Arlequin v3.5 [37]. Neutrality tests (Tajima’s D) were also performed to infer demographic history and deviations from neutral evolution.

## 3. Results

### 3.1. Genetic Clustering Characteristics of D. citri Based on Mitochondrial Genomes

In total, 133 mitochondrial genomes of *D. citri* were assembled and analyzed, including 77 previously published sequences, 13 newly assembled genomes from Hunan Province, and 43 metagenome-assembled mitogenomes (Table S1). The mitochondrial genomes had an average length of 15,020 bp and an average GC content of 25.50%, representing the largest *D. citri* mitochondrial genome dataset reported to date. Partitioned maximum-likelihood analysis of the complete mitochondrial genomes (Section 2.4) revealed two distinct major mitochondrial lineages: an Asian lineage and an American lineage (Figure 1), consistent with previous clustering analyses based on the *COI* gene [14,15,16]. A sensitivity analysis excluding the reference sequence MF426268.1 yielded essentially identical diversity estimates (184 polymorphic sites, 68 haplotypes, Hd = 0.9375, π = 0.001736), confirming that the results are robust to reference-sequence inclusion.

Within the Asian mitochondrial lineage, two distinct subgroups were evident. Eight samples from Yunnan, Sichuan, and Guizhou provinces clustered together as a monophyletic group with moderate statistical support (SH-aLRT = 73.1%, UFBoot = 47%). These sampling locations were all situated in the Yunnan–Guizhou Plateau region (Figure S2), with an average elevation of 1166 m (Table S2), forming a significant topographical contrast with lowland areas in Hunan, Guangdong, Zhejiang, Fujian, Guangxi, Hainan and Jiangxi provinces. The elevational barrier and associated climatic differences may have led to population isolation through restricted gene flow.

In contrast, samples from Hunan, Guangdong, Zhejiang, Fujian, Guangxi, Hainan and Jiangxi provinces, along with specimens from four Southeast Asian countries (Cambodia, Vietnam, Indonesia, and Malaysia), Africa (Kenya and Réunion Island), and Uruguay, formed a large, well-supported star-like cluster without intercontinental long branches (Figure 1). This pattern suggests that these geographically disparate samples may share a recent common ancestor, consistent with recent human-mediated dispersal from a common source population.

### 3.2. High Mitochondrial Genetic Differentiation Between the Major D. citri Mitochondrial Lineages

**Figure 2:**
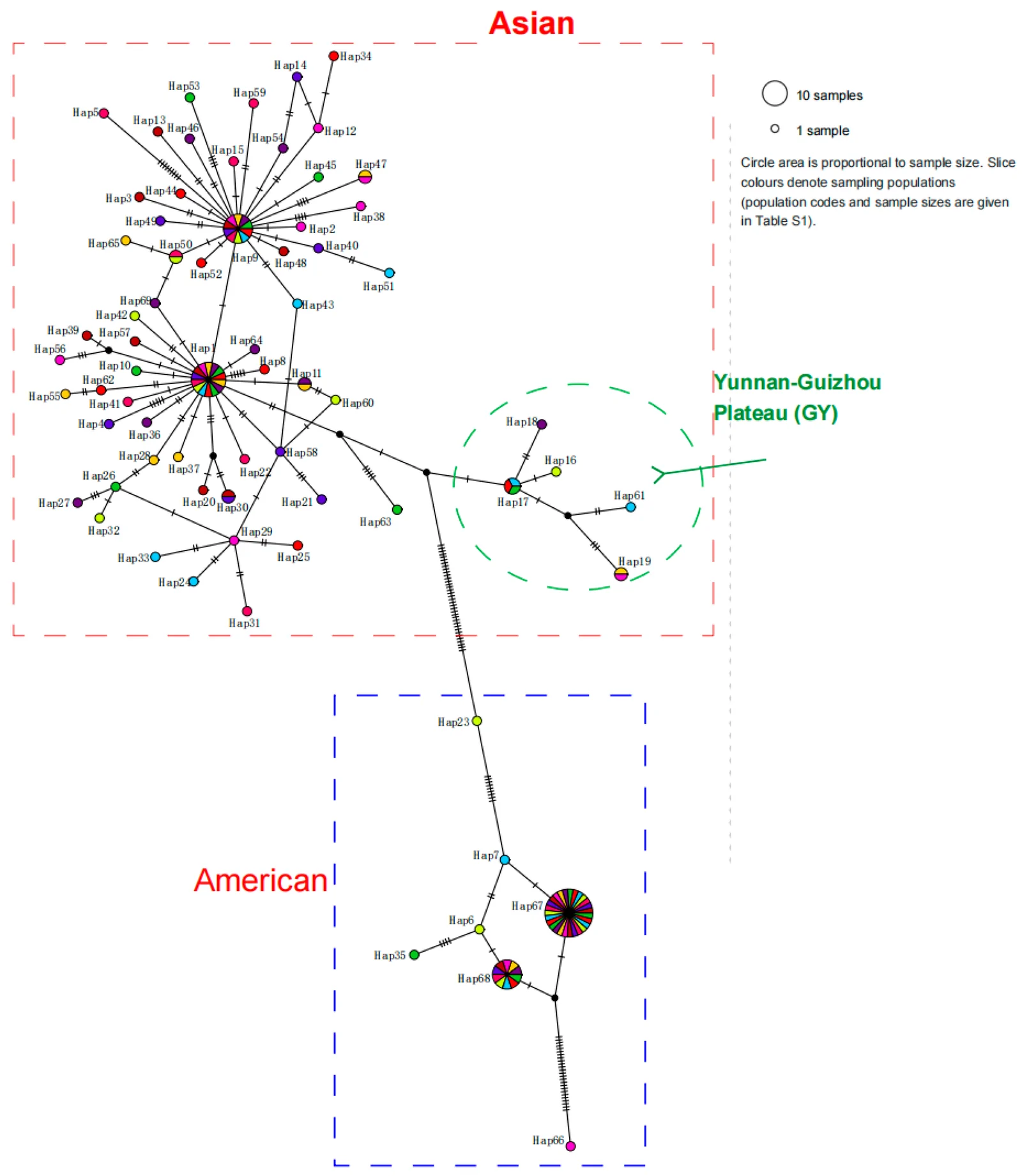
Median-joining haplotype network of 133 *Diaphorina citri* mitochondrial genomes showing 69 distinct haplotypes. Circle area is proportional to sample size; slice colors denote the sampling populations (one color per population; population codes and sample sizes are given in Table S1). Small black circles represent inferred median vectors, and hash marks on branches indicate the number of mutational steps between haplotypes. The green dashed ellipse marks the Yunnan–Guizhou Plateau (GY) samples. The network reveals a star-like structure emanating from central Asian-lineage haplotypes.

Population genetic analysis based on complete mitochondrial genomes of 133 individuals revealed 185 genetic variation sites, yielding 69 distinct haplotypes (Figure 2). Functional annotation and detailed distribution analysis of the 185 SNPs showed that 124 SNPs (67.0%) were located in protein-coding sequences and 61 SNPs (33.0%) in non-coding regions. The coding-region SNPs were distributed across 13 mitochondrial genes, with the highest densities in *CYTB* (17 SNPs, 13.7%), *ND5* (17 SNPs, 13.7%) and *COX1* (16 SNPs, 12.9%). Functionally, 51.6% of coding-region SNPs (64 SNPs) were in Complex I (NADH dehydrogenase), 24.2% (30 SNPs) in Complex IV (cytochrome c oxidase), 13.7% (17 SNPs) in Complex III (cytochrome bc1 complex) and 10.5% (13 SNPs) in ATP synthase, all of which are core components of mitochondrial energy metabolism (Table S4). Haplotype diversity (Hd) was high at 0.938 (variance = 0.00018, s.d. = 0.013), whereas nucleotide diversity (π) was relatively low at 0.00173. An exclusion sensitivity analysis without the reference genome MF426268.1 (132 genomes) yielded 184 polymorphic sites and 68 haplotypes, with haplotype diversity and nucleotide diversity essentially unchanged (Hd = 0.9375, π = 0.001736); all 32 fixed differences between the Asian and American lineages were recovered.

**Figure 3:**
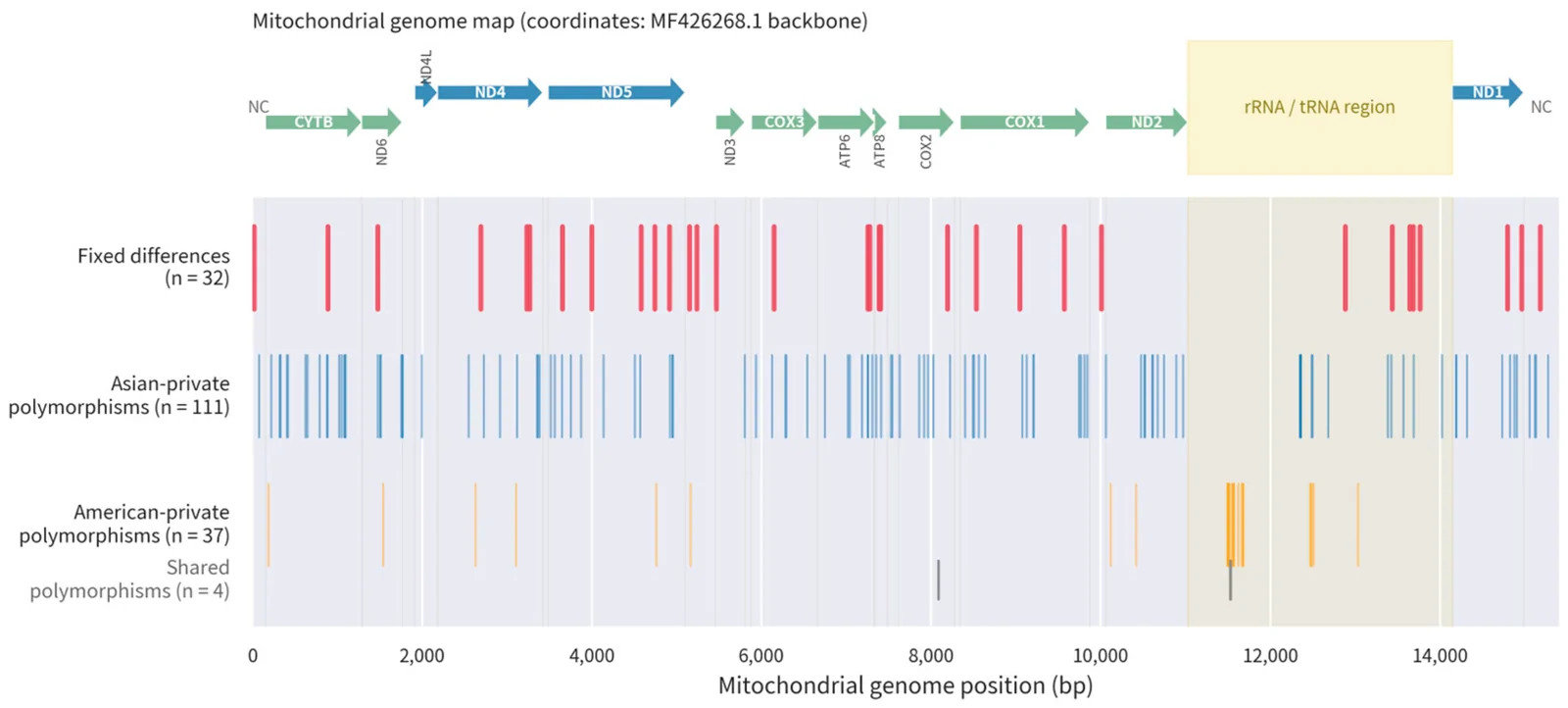
Distribution of fixed nucleotide differences and lineage-specific polymorphisms between the Asian and American mitochondrial lineages across the mitochondrial genome (coordinates refer to the MF426268.1 backbone, 15,400 bp). Top panel: gene map (blue arrows, + strand; green arrows, − strand; yellow shading, rRNA/tRNA region). Red ticks indicate the 32 fixed differences between the two lineages; blue and orange ticks indicate private polymorphisms of the Asian (*n* = 111) and American (*n* = 37) lineages, respectively; gray ticks indicate the four shared polymorphic sites. Variant counts refer to the 132-genome dataset excluding the reference sequence MF426268.1.

Mitogenome-wide SNP analysis revealed that *D. citri* can be divided into two highly differentiated mitochondrial lineages: an Asian lineage (*n* = 91, including Uruguay) and an American lineage (*n* = 42, including Pakistan) (Figure 2). AMOVA revealed that 56.03% of the total genetic variation was distributed between lineages (FST = 0.560, *p* < 0.001), with 43.97% within lineages. This differentiation was corroborated by 32 completely fixed SNP differences between the two lineages (Figure 3 and Table S5).

Functional annotation showed that the 32 fixed nucleotide differences (132 genomes, excluding the reference sequence MF426268.1) between the Asian and American mitochondrial lineages were distributed in both protein-coding genes and non-coding regions (Table S5). Of these fixed variants, seven were nonsynonymous substitutions located in mitochondrial respiratory-chain genes (*ND5*, *ND6*, *ATP8*, *COX2*, *COX1* and *ND1*), 15 were synonymous substitutions, and the remaining ten were located in non-coding RNA genes or intergenic regions. These fixed sites represent candidate lineage-specific molecular markers and indicate long-term genetic isolation between the two mitochondrial lineages.

At the mitogenome-wide level, excluding the reference genome MF426268.1 (132 genomes), the Asian mitochondrial lineage exhibited 111 unique polymorphic sites (polymorphic in the Asian lineage but fixed in the American lineage), whereas the American mitochondrial lineage possessed 37 unique polymorphic sites, with four shared polymorphic mutations between the two lineages (Table S6). These unique polymorphisms occurred in both coding and non-coding regions (Table S6), further supporting distinct evolutionary histories and limited genetic connectivity between the two lineages. The asymmetric distribution of private variants likely reflects differences in demographic history, lineage age and founder events during global colonization.

### 3.3. Significant Genetic Differentiation Between Yunnan–Guizhou Plateau and Lowland Populations

Within the Asian lineage, the Yunnan–Guizhou Plateau population (GY, *n* = 8) and the lowland plains population (PY2, *n* = 83, including SC, SA, Kenya, Réunion Island and Uruguay) exhibited significant genetic differentiation. Analysis of molecular variance (AMOVA) detected a moderate but significant genetic structure between the two groups, with 12.97% of the total genetic variation partitioned between plateau and plains populations (FST = 0.130, *p* < 0.05), while the majority (87.03%) occurred within populations. When the two plateau-collected samples carrying plains-type haplotypes (Changshun and Leibo; Section 2.6) were forcibly included in GY (*n* = 10) in a sensitivity analysis, differentiation remained highly significant (FST = 0.110, *p* < 0.001), supporting the robustness of the network-based grouping.

Despite the relatively small sample size, the GY population displayed lower nucleotide diversity (π = 0.00025) compared to the plains group (π = 0.00039) and harbored fewer unique variants (only 15 sites polymorphic in the plateau but monomorphic in the plains; Table S7), consistent with a smaller effective population size or stronger purifying selection in the highland lineage. Across the mitochondrial genome, 124 variable sites were identified within Asian individuals, of which 91 were located in protein-coding genes and 33 in tRNA genes, rRNA genes and intergenic regions (Table S7).

Neutrality tests yielded a significantly negative Tajima’s D value (D = −2.48872, *p* < 0.01) for the combined Asian dataset, indicating an excess of low-frequency variants consistent with recent demographic expansion or purifying selection. Two fixed nucleotide differences were identified in *trnG* and *rrn16* between the plateau and plains populations. Both fixed variants occur in non-coding RNA genes and do not result in amino acid changes. They may influence RNA secondary structure, stability or regulatory functions and are therefore candidate diagnostic markers for distinguishing the two lineages.

**Figure 4:**
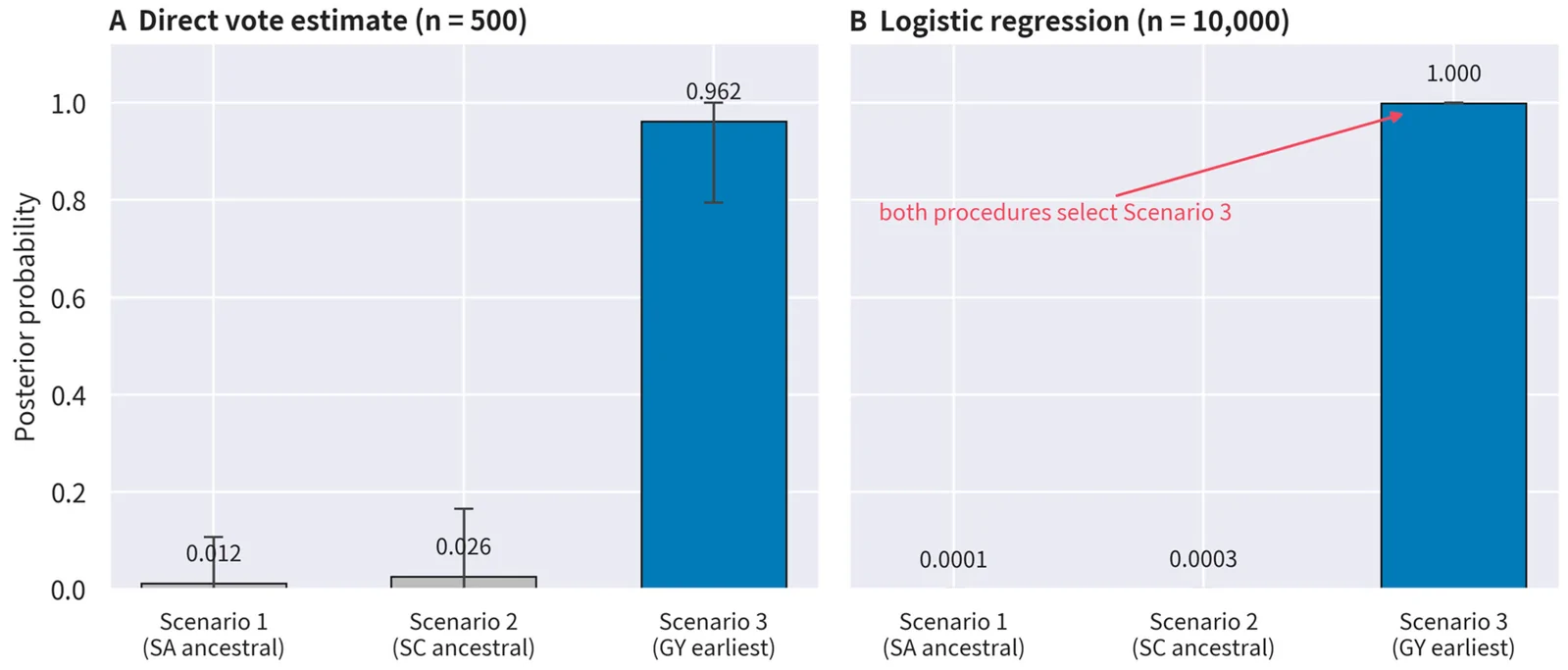
Model selection among three demographic scenarios for the Asian-lineage populations (DIYABC v2.0; widened N1 prior of 1 × 10^5^–1 × 10^7^). Posterior probabilities were estimated by (**A**) the direct approach (500 simulated datasets closest to the observed data) and (**B**) logistic regression (10,000 closest datasets). Both procedures concordantly favored Scenario 3 (GY as the earliest-diverging lineage, with SC and SA forming a sister group): direct approach *p* = 0.962 (95% CI 0.794–1.000); logistic regression *p* = 0.9995 (95% CI 0.9993–0.9998).

To further evaluate the historical divergence between these groups, Approximate Bayesian Computation (ABC) was performed using DIYABC v2.0 [34,35], comparing three competing demographic scenarios among Southeast Asia (SA), Southeast China (SC), and the Yunnan–Guizhou Plateau (GY) (Table S8). Under the widened N1 prior (1 × 10^5^–1 × 10^7^; Section 2.6), both model-choice procedures concordantly favored Scenario 3, in which GY is the earliest-diverging lineage and SC and SA form a more recent sister group (direct approach: posterior probability = 0.962, 95% CI 0.794–1.000; logistic regression: *p* = 0.9995, 95% CI 0.9993–0.9998; Figure 4). Parameter estimates under Scenario 3 (Table S9, Figure S1) showed that the effective population size of GY (N1) was not identifiable, with its posterior distribution increasing monotonically towards the upper prior bound, whereas N2 (SC) had a posterior mode of 5.07 × 10^4^ (95% CI 1.69 × 10^4^–9.74 × 10^4^) and N3 (SA) was only weakly identifiable. The divergence-time estimates were t1 = 3.52 × 10^4^ generations (GY–SC divergence; 95% CI 8.17 × 10^3^–4.90 × 10^4^) and t2 = 2.44 × 10^4^ generations (SC–SA divergence; 95% CI 1.29 × 10^4^–7.38 × 10^4^).

Taken together, the significant FST value, reduced nucleotide diversity in the plateau, two fixed non-coding RNA variants and moderately supported monophyletic clustering (SH-aLRT = 73.1%, UFBoot = 47%) provide consistent evidence that GY is a genetically distinct and historically isolated lineage. These results support the Yunnan–Guizhou Plateau as an important topographic barrier associated with long-term genetic isolation, although the exact divergence history requires further validation.

### 3.4. Global Population Genetic Structure and Dispersal Patterns

To address sampling imbalance, quantitative pairwise comparisons and TreeMix analysis were restricted to the five populations with *n* ≥ 4—Southeast Asia (SA, *n* = 4), Southeast China (SC, *n* = 67), Yunnan–Guizhou Plateau (GY, *n* = 8), North America (NA, *n* = 41), and Africa (AF, *n* = 11)—as described in the Materials and Methods. Pakistan and Uruguay (*n* = 1 each) were excluded from these analyses.

Pairwise population genetic analyses further supported the distinction between the Asian and American mitochondrial lineages (Table 1). Within the Asian mitochondrial lineage, the Yunnan–Guizhou Plateau (GY) and lowland (PY2) groups exhibited statistically significant differentiation (FST = 0.1297, *p* < 0.05). The nucleotide diversity of GY was substantially lower (π = 0.00025) than that of lowland populations (π = 0.00039). Although African samples were assigned to the Asian mitochondrial lineage, geographic pairwise comparisons showed differentiation between AF and SC/SA (FST = 0.353–0.399). These findings indicate that mitochondrial lineage assignment and geographic population structure are related but not identical.

## 4. Discussion

This study provides the most comprehensive analysis of *D. citri* mitochondrial genetic diversity to date, assembling 133 complete mitochondrial genomes and demonstrating substantial differentiation between two major mitochondrial lineages (FST = 0.560, *p* < 0.001). The observed differentiation exceeds that reported in previous studies using single mitochondrial genes (primarily *COI*), which detected genetic structure among populations from South America, North America, India, Iran, and Pakistan [14,15,16,22]. The whole-mitogenome approach revealed 32 fixed lineage-specific variants (132-genome dataset) and allowed lineage labels to be distinguished from sampling geography: Uruguay was placed within the Asian mitochondrial lineage, whereas Pakistan was placed within the American mitochondrial lineage.

The 32 fixed differences (132-genome dataset) between the Asian and American mitochondrial lineages represent candidate diagnostic markers within the sampled range. Seven nonsynonymous substitutions in mitochondrial respiratory-chain genes may have functional consequences, but adaptive significance cannot be inferred without protein functional assays, selection analyses and broader population validation. The plateau-specific variants in *trnG* and *rrn16* are non-coding RNA variants and likewise should be regarded as candidate markers rather than established adaptive substitutions.

The high haplotype diversity (Hd = 0.938) coupled with relatively low nucleotide diversity (π = 0.00173) observed in our dataset is consistent with recent population expansion following a bottleneck or founder event [38]. This pattern suggests that *D. citri* populations have undergone demographic expansion, generating numerous haplotypes through recent mutations, but insufficient time has elapsed for substantial sequence divergence to accumulate. Similar patterns have been documented in other invasive insect species and are characteristic of populations that have expanded rapidly from limited genetic diversity [39,40].

The Yunnan–Guizhou Plateau (GY) population exhibited statistically significant genetic differentiation from lowland populations (FST = 0.1297), markedly reduced nucleotide diversity (π = 0.00025), and a moderately supported monophyletic cluster (SH-aLRT = 73.1%, UFBoot = 47%; Figure 1). Two fixed differences in *trnG* and *rrn16* further distinguished all eight GY samples from 83 lowland samples. Under the widened N1 prior, both DIYABC model-choice procedures concordantly favored a scenario in which GY diverged earliest (Section 3.3), although the effective population size of GY (N1) was not identifiable from the present data. GY is therefore interpreted as a distinct and historically isolated lineage, while the exact timing of its divergence remains uncertain. Notably, two samples collected on the plateau (Changshun and Leibo) carried plains-type mitogenomes, indicating recent human-mediated movement of lowland psyllids into the highland region, likely via the citrus nursery trade, and underscoring the complex population dynamics of the Yunnan–Guizhou Plateau.

The reduced diversity in GY is consistent with a small effective population size, long-term isolation and/or historical bottlenecks. The rugged topography and ecological heterogeneity of the Yunnan–Guizhou Plateau may limit dispersal and maintain population subdivision. These processes can preserve lineage-specific mitochondrial variants even in the absence of demonstrated adaptive selection. The significantly negative Tajima’s D in the broader Asian dataset is compatible with recent demographic expansion or purifying selection and does not by itself demonstrate high-altitude adaptation.

Accordingly, the present data do not support claims of high-altitude adaptive evolution. The *trnG* and *rrn16* variants may affect RNA folding, stability or regulation, but these hypotheses require structure prediction, expression analysis and physiological validation. Future work should combine expanded plateau sampling, nuclear genomic data, formal selection tests and reciprocal-transplant or physiological experiments to determine whether plateau-associated differentiation has adaptive components.

Southeast China exhibited the highest haplotype diversity (Hd = 0.930), the largest number of private polymorphisms (*n* = 114), and signatures of recent demographic expansion. These patterns are compatible with Southeast China functioning as a post-colonization expansion center. However, Southeast Asia was represented by only four samples, so the data cannot exclude an incompletely sampled Southeast Asian source; we therefore refrain from designating a definitive primary global source population.

**Figure 5:**
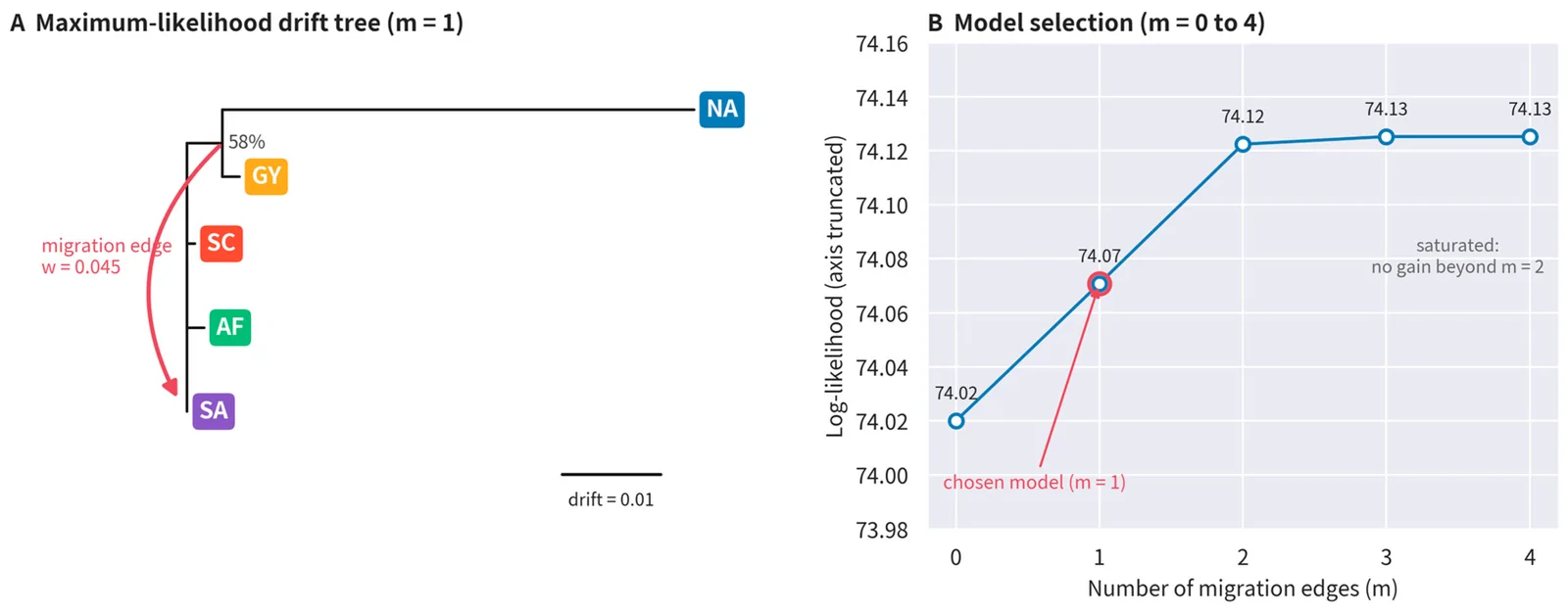
Maximum-likelihood drift tree of the five genetically assigned *Diaphorina citri* populations inferred by TreeMix from 1219 biallelic mitochondrial loci (131 mitogenomes; see Section 3.4). (**A**) Drift tree under the optimal m = 1 model; branch lengths are proportional to the drift parameter. The GY + NA grouping was recovered in 58% of 100 bootstrap replicates, and the exact main-analysis topology in 27%. The single migration edge (arrow; weight = 0.045) connects the branch ancestral to GY + NA to SA and is shown for completeness only; it was unstable across sample compositions and bootstrap replicates (Section 3.4). (**B**) Log-likelihood across m = 0–4 (note the truncated y-axis), showing saturation beyond m = 1.

TreeMix analysis provided complementary but limited evidence regarding historical connectivity (Figure 5). The maximum-likelihood drift tree placed SA as the earliest-diverging lineage, followed by AF, with SC sister to a GY + NA grouping. The GY + NA grouping (recovered in 58% of bootstrap replicates; the exact main-analysis topology in 27%) likely reflects the documented Asian origin of North American *Diaphorina citri* introductions rather than plateau-specific divergence, and the long NA branch (drift parameter = 0.048) is consistent with the predominantly laboratory-colony origin of that material (Section 2.1); the NA private-variant counts and divergence estimates involving NA should therefore be interpreted with caution. The single migration edge under the optimal m = 1 model (log-likelihood gain of only 0.05 over m = 0; weight = 0.045) connected the branch ancestral to GY + NA to SA; the inferred edge changed with sample composition (an earlier analysis with a different sample composition placed an edge between SC and GY), and only 4 of 10 time-limited m = 1 bootstrap replicates converged. We therefore treat the inferred edge strictly as a covariance signal and draw no directional gene-flow conclusions from it. The 45 shared polymorphisms between SC and NA likely reflect historical connectivity or retained ancestral variation, but the direction and timing cannot be resolved with the present dataset.

The single Pakistan sample was placed within the American mitochondrial lineage, whereas the single Uruguay sample was placed within the Asian mitochondrial lineage, demonstrating that geographic origin and mitochondrial ancestry do not always coincide. These placements may reflect modern trade-associated translocations, but each is based on only one sample and cannot support reconstruction of specific dispersal routes. The African samples showed affinity with Asian haplotypes, but their limited number (*n* = 11) also precludes robust inference of trans-oceanic pathways.

The lineage-specific variants identified here have potential applications in pest surveillance and biosecurity. The 32 fixed variants (132-genome dataset) distinguishing the two major mitochondrial lineages could be developed into molecular assays for population-origin assignment, while the *trnG* and *rrn16* variants could assist identification of the GY lineage. Such markers may support quarantine screening, tracing of new introductions and regional monitoring of *D. citri* movement. Before operational use, the markers should be validated using broader geographic sampling and assay-level testing, for example through qPCR-, ddPCR- or amplicon-based genotyping.

## 5. Conclusions

This study presents the largest *D. citri* mitochondrial-genome dataset assembled to date. The main conclusions are (1) global *D. citri* mitogenomes separate into two highly differentiated mitochondrial lineages, with Uruguay placed in the Asian lineage and Pakistan in the American lineage; (2) 32 fixed variants (132-genome dataset) between these lineages provide candidate markers for population-origin assignment, pending broader validation; (3) the Yunnan–Guizhou Plateau population is a genetically distinct and historically isolated lineage characterized by reduced diversity, moderately supported monophyly and two fixed non-coding RNA variants; and (4) Southeast China shows signatures consistent with post-colonization expansion, although limited Southeast Asian sampling prevents definitive reconstruction of global dispersal directionality.

Future studies should prioritize denser sampling from Southeast Asia, Pakistan, Uruguay and Africa, combined with nuclear genome sequencing and coalescent-based migration models. Functional assays are required to evaluate the biological effects of fixed coding and RNA variants, and broader assay-level validation is needed before candidate markers can be implemented in quarantine or routine pest-management programs.

## Figures and Tables

**Table 1:** Selected pairwise population genetic comparison results showing sample sizes, fixed differences, shared mutations, private mutation ratios, nucleotide diversity (π), absolute divergence (Dxy) and net genetic differentiation (Da) between retained geographic population pairs (*n* ≥ 4). Pakistan and Uruguay were excluded from quantitative comparisons. A dash (–) indicates a value that was not calculated or not applicable. *n*_1_ and *n*_2_ denote the sample sizes of population 1 and population 2; fixed differences are sites fixed for different alleles between the two populations; shared mutations are polymorphic sites shared by both populations (a value of 0 indicates no shared polymorphism); the private mutation ratio gives the numbers of private polymorphisms in population 1 versus population 2.

Comparison	*n*_1_	*n*_2_	Fixed Differences	Shared Mutations	Private Mutation	π1	π2	Dxy	Da
SA vs. SC	4	67	0	4	114:8	0.00057	0.00036	–	0.00001
SA vs. GY	4	8	4	5	11:11	0.00054	0.00025	0.00064	0.00027
SA vs. Africa	4	11	0	0	9:18	0.00053	0.00047	0.0007	0.0002
SC vs. Africa	67	11	0	0	18:18	0.00036	0.0004	–	0.00017
SC vs. NA	67	41	0	45	61:39	0.00036	0.00048	0.00337	0.00297

## Data Availability

The assembled mitochondrial genomes have been deposited in the China National GeneBank DataBase (CNGBdb) under BioProject accession PRJCA055168. Individual accession numbers and sequencing metadata are provided in Table S1.

## Supplementary Materials

The following supporting information can be downloaded at: https://www.mdpi.com/article/10.3390/insects17090917/s1, Tables S1–S10 and Figures S1–S3. Table S1. Detailed information for 133 *D. citri* mitochondrial genome samples, including GenBank accession numbers, collection location (country, province/state, specific locality), geographic coordinates, collection date, mitochondrial genome length, GC content, and haplotype assignment. Table S2. Elevation data for eight sampling sites in the Yunnan–Guizhou Plateau region, including province, specific locality, GPS coordinates (latitude and longitude), and elevation (m). Table S3. Assembly quality metrics for 43 metagenome-assembled *D. citri* mitochondrial genomes; Table S4. Functional annotation and genomic distribution of 185 mitochondrial SNPs detected across 133 *D. citri* individuals; Table S5. Thirty-two fixed nucleotide differences between the Asian and American *Diaphorina citri* mitochondrial lineages (132-genome dataset excluding the reference sequence MF426268.1): genomic positions (MF426268.1 backbone), gene annotations and functional classification. Table S6. Population-specific (private) polymorphic sites and allele frequencies in the Asian and American *D. citri* mitochondrial lineages; Table S7. Nucleotide variants and allele frequency differences between the Yunnan–Guizhou Plateau and lowland plains populations of *D. citri*; Table S8. Three competing demographic scenarios tested by DIYABC for inferring divergence history among Southeast Asia, Southeast China, and Yunnan–Guizhou Plateau populations of *D. citri*; Table S9. Prior distributions and posterior estimates of demographic parameters under DIYABC Scenario 3, which was concordantly favored by both the direct and logistic regression model-choice procedures; N1 was not identifiable (posterior increasing towards the upper prior bound). Table S10. Likelihood-based model comparison for TreeMix analysis of five genetically assigned populations of *Diaphorina citri* (131 mitogenomes, 1219 biallelic loci); log-likelihood saturated beyond m = 1, and the single m = 1 edge is interpreted as a covariance signal only. Figure S1. Prior and posterior distributions of demographic parameters estimated under Scenario 3 with the widened N1 prior (1 × 10^5^–1 × 10^7^). Figure S2. Geographic distribution of sampling locations in the Yunnan–Guizhou Plateau region, based on 90 m resolution digital elevation model data (site elevations 570–1684 m, mean 1166 m). Figure S3. Bootstrap validation of the TreeMix population drift tree in *Diaphorina citri* (100 replicates under m = 0; convergence tests under m = 1).
