# Supplementary material for: Mitochondrial Genomes Reveal Global Population Structure, Lineage Divergence, and Candidate Diagnostic Markers of Diaphorina citri
Source: Insects. 2026 Sep 1;17(9):917. doi: 10.3390/insects17090917 (PMC13607012; doi:10.3390/insects17090917)
Supplement: Supplementary file 1 [file insects-17-00917-s001.zip › insects-4526190-supplementary.pdf]

Supplementary Materials

Mitochondrial genomes reveal global population structure, lineage divergence, and candidate diagnostic markers of *Diaphorina citri*

Lijuan Yin, Suming Dai, Dan Wei, Dazhi Li and Yunsheng Wang

Table S1. Detailed information for 133 *D. citri* mitochondrial genome samples including GenBank accession numbers, collection location (country, province/state, specific locality), geographic coordinates, collection date, mitochondrial genome length, GC content, and haplotype assignment.

Accession	source	isolate	country	province	city	Longitude (°)	Latitude (°)	length (bp)	GC (%)	Haplotype	BioProject	Population assignment
MF426268.1	NCBI	DP2	China	Unknown	Unknown	NA	NA	15400	24.9	Hap10	—	SC
PP048992.1	NCBI	TWNT	China	Taiwan	Nantou	120.7	23.91	14978	25.55	Hap58	—	SC
PP048971.1	NCBI	GDCZ	China	Guangdong	Chaozhou	116.63	23.66	14980	25.55	Hap9	—	SC
PP048994.1	NCBI	ZJWZ	China	Zhejiang	Wenzhou	120.71	28	14978	25.55	Hap60	—	SC
PP048981.1	NCBI	GDQY	China	Guangdong	Qingyuan	113.06	23.69	14940	25.62	Hap9	—	SC
PP048975.1	NCBI	GDYN	China	Guangdong	Yangjiang	111.99	21.86	14989	25.56	Hap45	—	SC
PP048987.1	NCBI	GXGL	China	Guangxi	Guilin	110.19	25.24	14978	25.56	Hap54	—	SC
PP048996.1	NCBI	GZCH	China	Guizhou	Changshun	106.46	26.03	14978	25.55	Hap62	—	SC
MF614826.1	NCBI	Vietnam	Vietnam	Thái Bình	Đông H'ng	106.365	20.53	15024	25.45	Hap1	—	SA
MF614806.1	NCBI	GDJYpsy	China	Guangdong	Jieyang	116.38	23.56	15009	25.52	Hap1	—	SC
PP048973.1	NCBI	GDSGRH	China	Guangdong	Shaoguan	113.6	24.82	14994	25.56	Hap43	—	SC
MG489916.1	NCBI	GDGZ	China	Guangdong	Guangzhou	113.27	23.14	14999	25.54	Hap1	—	SC
MF614823.1	NCBI	TWHsinchu	China	Taiwan	Hsinchu	120.98	24.81	15010	25.52	Hap1	—	SC
MF614811.1	NCBI	HNHK	China	Hainan	Haikou	110.21	20.05	14993	25.54	Hap12	—	SC
PP048982.1	NCBI	GDMM	China	Guangdong	Maoming	110.93	21.67	14984	25.57	Hap50	—	SC
PP048970.1	NCBI	GDJY	China	Guangdong	Jieyang	116.38	23.56	14981	25.55	Hap41	—	SC
MF614807.1	NCBI	FJXM	China	Fujian	Xiamen	118.1	24.49	15017	25.51	Hap1	—	SC
PP048986.1	NCBI	JXGZ	China	Jiangxi	Ganzhou	114.94	25.84	14979	25.56	Hap50	—	SC
MF614827.1	NCBI	Indonesia	Indonesia	Jawa Tengah	Klaten	110.3	7.35	15030	25.46	Hap22	—	SA
MF614804.1	NCBI	GDHZ	China	Guangdong	Huizhou	114.42	23.12	14998	25.54	Hap9	—	SC
MF614813.1	NCBI	GXNN	China	Guangxi	Nanning	108.37	22.82	14987	25.53	Hap14	—	SC
MF614803.1	NCBI	GDSG	China	Guangdong	Shaoguan	113.6	24.82	15011	25.52	Hap9	—	SC

Accession	source	isolate	country	province	city	Longitude (°)	Latitude (°)	length (bp)	GC (%)	Haplotype	BioProject	Population assignment
PP048990.1	NCBI	FJQZ	China	Fujian	Quanzhou	118.68	24.88	14993	25.5	Hap57	—	SC
PP048972.1	NCBI	GDST	China	Guangdong	Shantou	116.69	23.36	14979	25.57	Hap42	—	SC
NC_030214.1	NCBI	JXXF	China	jiangxi	xinfeng	114.93	25.39	14996	25.53	Hap34	—	SC
MF614814.1	NCBI	ZJpsy	China	Zhejiang	Taizhou	121.43	28.66	15009	25.53	Hap11	—	SC
MF614805.1	NCBI	GDYC	China	Guangdong	Yangchun	111.8	22.18	15011	25.53	Hap9	—	SC
PP048984.1	NCBI	JXFZ	China	Jiangxi	Fuzhou	111.8	22.18	14995	25.56	Hap52	—	SC
PP048969.1	NCBI	GDHZBL	China	Guangdong	Huizhou	114.42	23.12	14890	25.55	Hap40	—	SC
MF614809.1	NCBI	JXJA	China	Jiangxi	Ji'an	115	27.12	15010	25.53	Hap9	—	SC
MF614808.1	NCBI	FJFZ	China	Fujian	Fuzhou	119.3	26.08	15012	25.53	Hap11	—	SC
MF614810.1	NCBI	JXGC	China	Jiangxi	Guangchang	116.34	26.85	15012	25.53	Hap9	—	SC
PP048977.1	NCBI	GDMZ	China	Guangdong	Meizhou	116.13	24.29	14979	25.55	Hap46	—	SC
PP048968.1	NCBI	GDFS	China	Guangdong	Foshan	113.13	23.03	14992	25.54	Hap39	—	SC
PP048993.1	NCBI	HNYX	China	Hunan	Yongxing	113.12	26.13	14979	25.56	Hap59	—	SC
PP048979.1	NCBI	GDJM	China	Guangdong	Jiangmen	113.09	22.58	14993	25.56	Hap48	—	SC
MF614815.1	NCBI	HNYZ	China	Hunan	Yizhang	112.96	25.41	15008	25.52	Hap15	—	SC
PP048980.1	NCBI	GDZQ	China	Guangdong	Zhaoqing	112.47	23.05	14979	25.57	Hap49	—	SC
MF614812.1	NCBI	GXYL	China	Guangxi	Yulin	110.19	22.66	14992	25.53	Hap13	—	SC
PP048967.1	NCBI	GDGZCH	China	Guangdong	Guangzhou	113.59	23.55	14978	25.54	Hap38	—	SC
PP048985.1	NCBI	JXPX	China	Jiangxi	Pingxiang	113.89	27.66	14978	25.55	Hap53	—	SC
MF614825.1	NCBI	Malaysia	Malaysia	Pahang	Kuntan	103.326	3.8077	15027	25.47	Hap21	—	SA
PP048974.1	NCBI	GDYF	China	Guangdong	Yunfu	112.05	22.92	14999	25.6	Hap44	—	SC
PP048983.1	NCBI	GDZJ	China	Guangdong	Zhanjiang	110.37	21.28	14990	25.56	Hap51	—	SC
PP048988.1	NCBI	GXQZ	China	Guangxi	Qinzhou	108.66	21.99	14976	25.59	Hap55	—	SC
MF614820.1	NCBI	YNJS	China	Yunnan	Jianshui	102.83	23.64	15001	25.48	Hap17	—	GY
MF614824.1	NCBI	Cambodia	Cambodia	Siem Reap	Krong	103.8603	13.364	15009	25.54	Hap20	—	SA
MF614817.1	NCBI	SCNN	China	Sichuan	Ningnan	102.76	27.07	14999	25.48	Hap16	—	GY
PP048989.1	NCBI	GXWZ	China	Guangxi	Wuzhou	111.29	23.48	14993	25.54	Hap56	—	SC

Accession	source	isolate	country	province	city	**Longitude (°)**	**Latitude (°)**	length (bp)	GC (%)	Haplotype	**BioProject**	**Population assignment**
MF614819.1	NCBI	YNBC	China	Yunnan	Binchuan	100.58	25.84	14999	25.48	Hap16	—	GY
PP048978.1	NCBI	GDHY	China	Guangdong	Heyuan	114.71	23.75	14985	25.57	Hap47	—	SC
PP048991.1	NCBI	HNCM	China	Hunan	Chenzhou	113.02	25.78	14980	25.55	Hap47	—	SC
MF614816.1	NCBI	SCMY	China	Sichuan	Miyi	102.12	26.9	15014	25.51	Hap16	—	GY
PP048995.1	NCBI	YNMS	China	Yunnan	Mengzi	103.37	23.4	14922	25.57	Hap61	—	GY
MF614818.1	NCBI	GZWM	China	Guizhou	Wangmo	106.11	25.18	15011	25.49	Hap18	—	GY
PP048997.1	NCBI	SCLB	China	Sichuan	Leibo	103.58	28.27	14980	25.54	Hap63	—	SC
MF614821.1	NCBI	YNRL	China	Yunnan	Ruili	97.86	24.02	15011	25.49	Hap19	—	GY
MF614822.1	NCBI	YNBS	China	Yunnan	Baoshan	99.17	25.12	15020	25.48	Hap19	—	GY
MF614828.1	NCBI	Pakistan	Pakistan	Khyber Pakhtunkhwa	Peshawar	71.5675	34.01444	15015	25.46	Hap23	—	not assigned
KY426014.1	NCBI	CA	USA	California	San Gabriel	-118.1058	34.0961	15027	25.43	Hap6	—	NA
KY426015.1	NCBI	FL	USA	Florida	Immokalee	-81.4173	26.4187	15012	25.49	Hap7	—	NA
MW021148.1	NCBI	RLPA	Réunion	La Réunion	Petite île	55.5592	-21.3411	15008	25.52	Hap26	—	AF
MW021151.1	NCBI	RLLA	Réunion	La Réunion	Le Tampon	55.5153	-21.2781	15008	25.51	Hap29	—	AF
MW021160.1	NCBI	RLPC	Réunion	La Réunion	Petite île	55.5592	-21.3411	15004	25.51	Hap33	—	AF
MW021147.1	NCBI	RLV	Réunion	La Réunion	Villèle	55.26375	-21.05597	15006	25.5	Hap25	—	AF
MW021158.1	NCBI	RLPD	Réunion	La Réunion	Pierrefond	55.4282	-21.3019	15007	25.51	Hap31	—	AF
MW021159.1	NCBI	RLLB	Réunion	La Réunion	Le Tampon	55.5153	-21.2781	15009	25.53	Hap32	—	AF
MW021150.1	NCBI	RLPB	Réunion	La Réunion	Petite île	55.5592	-21.3411	15010	25.54	Hap28	—	AF
MW021149.1	NCBI	RLLC	Réunion	La Réunion	Le Tampon	55.5153	-21.2781	15010	25.52	Hap27	—	AF
MW021146.1	NCBI	RLPE	Réunion	La Réunion	Pierrefond	55.4282	-21.3019	15009	25.51	Hap24	—	AF
KX073968.1	NCBI	JXNC	China	Jiangxi	Nanchang	115.86	28.69	15038	25.44	Hap5	—	SC
MW021155.1	NCBI	KKL	Kenya	Kwale	Lunga Lunga	39.1231	-4.555	15012	25.5	Hap30	—	AF
MW021152.1	NCBI	KKA	Kenya	Karicho	Awasi	35.0735	-0.16058	15012	25.51	Hap30	—	AF
SRR23454944	NCBI	DP1	China	Guangxi	Unknown	NA	NA	15112	26.38	Hap69	PRJNA933393	SC

Accession	source	isolate	country	province	city	Longitude (°)	Latitude (°)	length (bp)	GC (%)	Haplotype	BioProject	Population assignment
OM181947.1	NCBI	Uruguay	Uruguay	Unknown	Unknown	-57.75548	-31.287202	14965	25.57	Hap37	—	not assigned
OM181946.1	NCBI	CRF-TW	China	Taiwan	Unknown	120.67988	24.143171	15145	25.28	Hap36	—	SC
OM181945.1	NCBI	CRF-CA	USA	California	Unknown	-122.27139	37.80222	15038	25.45	Hap35	—	NA
AX	Lab	HNAX	China	Hunan	Anxiang	112.18	29.42	14986	25.56	Hap1	PRJCA055168	SC
CX	Lab	HNCX	China	Hunan	Chenxi	110.19	28.01	15001	25.52	Hap1	PRJCA055168	SC
DC	Lab	HNDC	China	Hunan	Dingcheng	111.69	29.02	14984	25.59	Hap3	PRJCA055168	SC
DK	Lab	HNDK	China	Hunan	Dongkou	110.58	27.07	14963	25.56	Hap4	PRJCA055168	SC
HY	Lab	HNHY	China	Hunan	Huayuan	109.49	28.58	14953	25.59	Hap1	PRJCA055168	SC
LST	Lab	HNLST	China	Hunan	Lengshuitan	111.6	26.47	14809	25.71	Hap8	PRJCA055168	SC
LX-hu	Lab	HNLX-hu	China	Hunan	Luxi	103.77	24.54	14945	25.6	Hap1	PRJCA055168	SC
LY-liu	Lab	HNLY-liu	China	Hunan	Liuyang	113.65	28.17	14949	25.61	Hap9	PRJCA055168	SC
LY	Lab	HNLY	China	Hunan	Lianyuan	111.67	27.7	14979	25.54	Hap1	PRJCA055168	SC
SF	Lab	HNSF	China	Hunan	Shuangfeng	112.18	27.46	14916	25.67	Hap9	PRJCA055168	SC
SM	Lab	HNSM	China	Hunan	Shimen	111.39	29.59	14930	25.65	Hap64	PRJCA055168	SC
XXBJ	Lab	HNXXBJ	China	Hunan	Baojing	109.67	28.71	14894	25.61	Hap1	PRJCA055168	SC
CZYZ	Lab	HNCZYZ	China	Hunan	Yizhang	112.96	25.41	14978	25.58	Hap2	PRJCA055168	SC
SRR16895067	NCBI	USAIA	USA	New York	Ithaca	-76.501884	42.443962	14609	25.888151	Hap66	PRJNA779156 (laborator	NA

Accession	source	isolate	country	province	city	Longitude (°)	Latitude (°)	length (bp)	GC (%)	Haplotype	BioProject	Population assignment
											y colony)	
SRR16895068	NCBI	USAIB	USA	New York	Ithaca	-76.501884	42.443962	15071	25.386504	Hap67	PRJNA779156 (laboratory colony)	NA
SRR16895070	NCBI	USAIC	USA	New York	Ithaca	-76.501884	42.443962	15031	25.454062	Hap67	PRJNA779156 (laboratory colony)	NA
SRR16895071	NCBI	USAID	USA	New York	Ithaca	-76.501884	42.443962	15033	25.450675	Hap67	PRJNA779156 (laboratory colony)	NA
SRR16895072	NCBI	USAIE	USA	New York	Ithaca	-76.501884	42.443962	15089	25.35622	Hap67	PRJNA779156 (laboratory colony)	NA
SRR16895073	NCBI	USAIF	USA	New York	Ithaca	-76.501884	42.443962	15154	25.280454	Hap67	PRJNA779156 (laboratory colony)	NA
SRR16895074	NCBI	USAI3	USA	New York	Ithaca	-76.501884	42.443962	15191	25.416365	Hap68	PRJNA779156 (laboratory colony)	NA
SRR16895075	NCBI	USAIG	USA	New York	Ithaca	-76.501884	42.443962	15015	25.481185	Hap67	PRJNA779156 (laboratory colony)	NA
SRR16895077	NCBI	USAIH	USA	New York	Ithaca	-76.501884	42.443962	15078	25.394615	Hap67	PRJNA779156 (laboratory colony)	NA
SRR16895078	NCBI	USAI4	USA	New York	Ithaca	-76.501884	42.443962	15112	25.628639	Hap68	PRJNA779156 (laboratory colony)	NA
SRR16895079	NCBI	USAI5	USA	New York	Ithaca	-76.501884	42.443962	15067	25.406518	Hap68	PRJNA779156 (laborator	NA

Accession	source	isolate	country	province	city	Longitude (°)	Latitude (°)	length (bp)	GC (%)	Haplotype	BioProject	Population assignment
											y colony)	
SRR16895080	NCBI	USAI6	USA	New York	Ithaca	-76.501884	42.443962	15125	25.368595	Hap68	PRJNA779156 (laboratory colony)	NA
SRR16895081	NCBI	USAI7	USA	New York	Ithaca	-76.501884	42.443962	15064	25.477961	Hap68	PRJNA779156 (laboratory colony)	NA
SRR16895082	NCBI	USAII	USA	New York	Ithaca	-76.501884	42.443962	15087	25.359581	Hap67	PRJNA779156 (laboratory colony)	NA
SRR16895083	NCBI	USAIJ	USA	New York	Ithaca	-76.501884	42.443962	15016	25.479489	Hap67	PRJNA779156 (laboratory colony)	NA
SRR16895084	NCBI	USAIK	USA	New York	Ithaca	-76.501884	42.443962	15086	25.361262	Hap67	PRJNA779156 (laboratory colony)	NA
SRR16895085	NCBI	USAIL	USA	New York	Ithaca	-76.501884	42.443962	15057	25.410108	Hap67	PRJNA779156 (laboratory colony)	NA
SRR16895087	NCBI	USAI8	USA	New York	Ithaca	-76.501884	42.443962	15064	25.491237	Hap68	PRJNA779156 (laboratory colony)	NA
SRR16895088	NCBI	USAIM	USA	New York	Ithaca	-76.501884	42.443962	15084	25.364625	Hap67	PRJNA779156 (laboratory colony)	NA
SRR16895089	NCBI	USAI9	USA	New York	Ithaca	-76.501884	42.443962	15136	25.343552	Hap68	PRJNA779156 (laboratory colony)	NA
SRR16895091	NCBI	USAIN	USA	New York	Ithaca	-76.501884	42.443962	15152	25.369588	Hap67	PRJNA779156 (laborator	NA

Accession	source	isolate	country	province	city	Longitude (°)	Latitude (°)	length (bp)	GC (%)	Haplotype	BioProject	Population assignment
											y colony)	
SRR16895092	NCBI	USAIO	USA	New York	Ithaca	-76.501884	42.443962	15160	25.395778	Hap67	PRJNA779156 (laboratory colony)	NA
SRR16895093	NCBI	USAIP	USA	New York	Ithaca	-76.501884	42.443962	15159	25.239132	Hap67	PRJNA779156 (laboratory colony)	NA
SRR16895094	NCBI	USAIQ	USA	New York	Ithaca	-76.501884	42.443962	15048	25.425306	Hap67	PRJNA779156 (laboratory colony)	NA
SRR16895095	NCBI	USAIR	USA	New York	Ithaca	-76.501884	42.443962	15057	25.410108	Hap67	PRJNA779156 (laboratory colony)	NA
SRR16895096	NCBI	USAI10	USA	New York	Ithaca	-76.501884	42.443962	15125	25.500826	Hap68	PRJNA779156 (laboratory colony)	NA
SRR16895099	NCBI	USAIS	USA	New York	Ithaca	-76.501884	42.443962	15064	25.398301	Hap67	PRJNA779156 (laboratory colony)	NA
SRR16895100	NCBI	USAIT	USA	New York	Ithaca	-76.501884	42.443962	15076	25.404617	Hap67	PRJNA779156 (laboratory colony)	NA
SRR16895101	NCBI	USAIU	USA	New York	Ithaca	-76.501884	42.443962	15059	25.406734	Hap67	PRJNA779156 (laboratory colony)	NA
SRR16895102	NCBI	USAIV	USA	New York	Ithaca	-76.501884	42.443962	15024	25.465921	Hap67	PRJNA779156 (laboratory colony)	NA
SRR16895103	NCBI	USAI11	USA	New York	Ithaca	-76.501884	42.443962	15013	25.471258	Hap68	PRJNA779156 (laborator	NA

**Accession**	**source**	**isolate**	**country**	**province**	**city**	**Longitude (°)**	**Latitude (°)**	**length (bp)**	**GC (%)**	**Haplotype**	**BioProject**	**Population assignment**
											y colony)	
SRR16895104	NCBI	USAIW	USA	New York	Ithaca	-76.501884	42.443962	15169	25.321379	Hap67	PRJNA779156 (laboratory colony)	NA
SRR16895105	NCBI	USAI12	USA	New York	Ithaca	-76.501884	42.443962	15083	25.406086	Hap68	PRJNA779156 (laboratory colony)	NA
SRR16895106	NCBI	USAIX	USA	New York	Ithaca	-76.501884	42.443962	15119	25.312521	Hap67	PRJNA779156 (laboratory colony)	NA
SRR16895108	NCBI	USAIY	USA	New York	Ithaca	-76.501884	42.443962	15085	25.38946	Hap67	PRJNA779156 (laboratory colony)	NA
SRR16895111	NCBI	USAIZ	USA	New York	Ithaca	-76.501884	42.443962	15356	25.234436	Hap67	PRJNA779156 (laboratory colony)	NA
SRR16895112	NCBI	USAI1	USA	New York	Ithaca	-76.501884	42.443962	15027	25.460837	Hap67	PRJNA779156 (laboratory colony)	NA
SRR16895113	NCBI	USAI2	USA	New York	Ithaca	-76.501884	42.443962	15062	25.401673	Hap67	PRJNA779156 (laboratory colony)	NA
SRR23454940	NCBI	GXGL1	China	Guangxi	Guilin	110.33	25.31	14993	25.545254	Hap1	PRJNA933393	SC
SRR23454942	NCBI	GXGL2	China	Guangxi	Guilin	110.33	25.31	14960	25.581551	Hap1	PRJNA933393	SC
SRR23454943	NCBI	GXGL3	China	Guangxi	Guilin	110.33	25.31	15226	25.59	Hap1	PRJNA933393	SC
SRR16559822	NCBI	China-M	China	Guangxi	Guilin	110.33	25.31	14910	25.64051	Hap9	PRJNA774108	SC
SRR16559823	NCBI	China-F	China	Guangxi	Guilin	110.33	25.31	14968	25.601283	Hap65	PRJNA77	SC

Accession	source	isolate	country	province	city	Longitude (°)	Latitude (°)	length (bp)	GC (%)	Haplotype	BioProject	Population assignment
											4108	

Table S2. Elevation data for eight sampling sites in the Yunnan-Guizhou Plateau region, including province, specific locality, GPS coordinates (latitude and longitude), elevation (m).

ID	Accession	Sample	Longitude (°)	Latitude (°)	height	Haplotype
1	MF614820.1	Jianshui	102.83	23.64	1334.47	Hap17
2	MF614817.1	Ningnan	102.76	27.07	1124.83	Hap16
3	MF614819.1	Binchuan	100.58	25.84	1431.67	Hap16
4	MF614816.1	Miyi	102.12	26.9	1083.52	Hap16
5	PP048995.1	Mengzi	103.37	23.4	1320.36	Hap61
6	MF614818.1	Wangmo	106.11	25.18	570.24	Hap18
7	MF614821.1	Ruili	97.86	24.02	783	Hap19
8	MF614822.1	Baoshan	99.17	25.12	1684.15	Hap19

Table S3. Assembly quality metrics for 43 metagenome-assembled *D. citri* mitochondrial genomes.

sample_id	assembly_length	num_contigs	longest_contig	n50	gc_content_pct	mean_depth	median_depth	depth_coverage_1x_pct	depth_coverage_5x_pct	depth_coverage_10x_pct	ref_identity_pct	ref_coverage_pct	aligned_bases	mismatches	gaps	is_circular	circular_overlap_bp	circular_overlap_identity_pct	length_vs_ref_ratio_pct
SRR16895067	14609	1	14609	14609	25.89	NA	NA	NA	NA	NA	99.62	95.26	14291	54	4	No	0	0	97.4
SRR16895068	15071	1	15071	15071	25.39	NA	NA	NA	NA	NA	99.44	100	15019	84	8	No	0	0	100.48
SRR16895070	15031	1	15031	15031	25.45	NA	NA	NA	NA	NA	99.44	99.85	14997	84	8	No	0	0	100.21
SRR16895071	15033	1	15033	15033	25.45	NA	NA	NA	NA	NA	99.44	99.99	15017	84	8	No	0	0	100.23
SRR16895072	15089	1	15089	15089	25.36	NA	NA	NA	NA	NA	99.42	100	15087	88	9	No	0	0	100.6
SRR16895073	15154	1	15154	15154	25.28	NA	NA	NA	NA	NA	99.42	100	15079	87	9	No	0	0	101.03
SRR16895074	15191	1	15191	15191	25.42	NA	NA	NA	NA	NA	99.47	100	15124	80	9	No	0	0	101.28
SRR16895075	15015	1	15015	15015	25.48	NA	NA	NA	NA	NA	99.44	99.85	14997	84	8	No	0	0	100.11
SRR16895077	15078	1	15078	15078	25.39	NA	NA	NA	NA	NA	99.44	100	15019	84	8	No	0	0	100.53
SRR16895078	15112	1	15112	15112	25.63	NA	NA	NA	NA	NA	99.43	99.87	15001	85	9	No	0	0	100.75
SRR16895079	15067	1	15067	15067	25.41	NA	NA	NA	NA	NA	99.49	100	15008	77	8	No	0	0	100.45
SRR16895080	15125	1	15125	15125	25.37	NA	NA	NA	NA	NA	99.47	99.85	14993	80	8	No	0	0	100.84
SRR16895081	15064	1	15064	15064	25.48	NA	NA	NA	NA	NA	99.51	99.85	14983	73	8	No	0	0	100.43
SRR16895082	15087	1	15087	15087	25.36	NA	NA	NA	NA	NA	99.45	100	15018	83	8	No	0	0	100.59
SRR16895083	15016	1	15016	1501	25.48	NA	NA	NA	NA	NA	99.45	99.85	14995	82	8	No	0	0	100.11

sample_id	assembly_length	num_contigs	longest_contig	n50	gc_content_pct	mean_depth	median_depth	depth_coverage_1x_pct	depth_coverage_5x_pct	depth_coverage_10x_pct	ref_identity_pct	ref_coverage_pct	aligned_bases	mismatches	gaps	is_circular	circular_overlap_bp	circular_overlap_identity_pct	length_vs_ref_ratio_pct
				6															
SRR16895084	15086	1	15086	15086	25.36	NA	NA	NA	NA	NA	99.43	100	15020	85	8	No	0	0	100.58
SRR16895085	15057	1	15057	15057	25.41	NA	NA	NA	NA	NA	99.45	99.97	15014	83	8	No	0	0	100.39
SRR16895087	15064	1	15064	15064	25.49	NA	NA	NA	NA	NA	99.44	99.85	14997	84	8	No	0	0	100.43
SRR16895088	15084	1	15084	15084	25.36	NA	NA	NA	NA	NA	99.45	100	15018	83	8	No	0	0	100.57
SRR16895089	15136	1	15136	15136	25.34	NA	NA	NA	NA	NA	99.41	100	15036	88	9	No	0	0	100.91
SRR16895091	15152	1	15152	15152	25.37	NA	NA	NA	NA	NA	99.45	99.99	15016	83	8	No	0	0	101.02
SRR16895092	15160	1	15160	15160	25.4	NA	NA	NA	NA	NA	99.45	100	15018	83	8	No	0	0	101.07
SRR16895093	15159	1	15159	15159	25.24	NA	NA	NA	NA	NA	99.41	100	15097	89	9	No	0	0	101.07
SRR16895094	15048	1	15048	15048	25.43	NA	NA	NA	NA	NA	99.45	99.98	15015	83	8	No	0	0	100.33
SRR16895095	15057	1	15057	15057	25.41	NA	NA	NA	NA	NA	99.45	100	15018	83	8	No	0	0	100.39
SRR16895096	15125	1	15125	15125	25.5	NA	NA	NA	NA	NA	99.46	100	15016	81	8	No	0	0	100.84
SRR16895099	15064	1	15064	15064	25.4	NA	NA	NA	NA	NA	99.45	100	15018	83	8	No	0	0	100.43
SRR16895100	15076	1	15076	15076	25.4	NA	NA	NA	NA	NA	99.38	100	15075	94	9	No	0	0	100.51
SRR16895101	15059	1	15059	15059	25.41	NA	NA	NA	NA	NA	99.45	100	15018	83	8	No	0	0	100.4
SRR16895102	15024	1	15024	15024	25.47	NA	NA	NA	NA	NA	99.44	99.89	15003	84	8	No	0	0	100.17

sample_id	assembly_length	num_contigs	longest_contig	n50	gc_content_pct	mean_depth	median_depth	depth_coverage_1x_pct	depth_coverage_5x_pct	depth_coverage_10x_pct	ref_identity_pct	ref_coverage_pct	aligned_bases	mismatches	gaps	is_circular	circular_overlap_bp	circular_overlap_identity_pct	length_vs_ref_ratio_pct
SRR16895103	15013	1	15013	15013	25.47	NA	NA	NA	NA	NA	99.51	99.82	14979	73	8	No	0	0	100.09
SRR16895104	15169	1	15169	15169	25.32	NA	NA	NA	NA	NA	99.24	100	15139	115	9	No	0	0	101.13
SRR16895105	15083	1	15083	15083	25.41	NA	NA	NA	NA	NA	99.45	99.97	15014	83	8	No	0	0	100.56
SRR16895106	15119	1	15119	15119	25.31	NA	NA	NA	NA	NA	99.42	100	15097	88	9	No	0	0	100.8
SRR16895108	15085	1	15085	15085	25.39	NA	NA	NA	NA	NA	99.45	100	15018	83	8	No	0	0	100.57
SRR16895111	15356	1	15356	15356	25.23	NA	NA	NA	NA	NA	99.43	100	15021	86	8	No	0	0	102.38
SRR16895112	15027	1	15027	15027	25.46	NA	NA	NA	NA	NA	99.45	99.89	15001	82	8	No	0	0	100.19
SRR16895113	15062	1	15062	15062	25.4	NA	NA	NA	NA	NA	99.43	100	15020	85	8	No	0	0	100.42
SRR23454940	14993	1	14993	14993	25.55	NA	NA	NA	NA	NA	99.86	99.85	14984	21	6	No	0	0	99.96
SRR23454942	14960	1	14960	14960	25.58	NA	NA	NA	NA	NA	99.91	99.61	14947	13	4	No	0	0	99.74
SRR23454943	15226	1	15226	15226	25.59	NA	NA	NA	NA	NA	99.93	99.19	14882	10	4	No	0	0	101.51
SRR16559822	14910	1	14910	14910	25.64	NA	NA	NA	NA	NA	99.93	99.35	14905	11	4	No	0	0	99.41
SRR16559823	14968	1	14968	14968	25.6	NA	NA	NA	NA	NA	99.89	99.76	14970	16	6	No	0	0	99.79

Table S4. Functional annotation and genomic distribution of 185 mitochondrial SNPs detected across 133 *D. citri* individuals.

Site	Region	Gene	Gene	Number of SNPs	Proportion of coding-region SNPs (%)
20	Coding sequence	ND1	CYTB	17	13.7
77	Non-coding region		ND5	17	13.7
186	Coding sequence	CYTB	COX1	16	12.9
224	Coding sequence	CYTB	ND1	12	9.7
322	Coding sequence	CYTB	ND4	12	9.7
330	Coding sequence	CYTB	ND2	11	8.9
409	Coding sequence	CYTB	ND6	9	7.3
417	Coding sequence	CYTB	ATP6	9	7.3
624	Coding sequence	CYTB	COX2	8	6.5
651	Coding sequence	CYTB	COX3	6	4.8
795	Coding sequence	CYTB	ATP8	4	3.2
882	Coding sequence	CYTB	ND3	2	1.6
888	Coding sequence	CYTB	ND4L	1	0.8
894	Coding sequence	CYTB			
1026	Coding sequence	CYTB			
1053	Coding sequence	CYTB			
1085	Coding sequence	CYTB			
1088	Coding sequence	CYTB			
1100	Coding sequence	CYTB			
1478	Coding sequence	ND6			
1480	Coding sequence	ND6			
1503	Coding sequence	ND6			
1508	Coding sequence	ND6			
1513	Coding sequence	ND6			
1544	Coding sequence	ND6			
1757	Coding sequence	ND6			
1764	Coding sequence	ND6			

Site	Region	Gene	Gene	Number of SNPs	Proportion of coding-region SNPs (%)
1770	Coding sequence	ND6			
1995	Coding sequence	ND4L			
2548	Coding sequence	ND4			
2633	Coding sequence	ND4			
2696	Coding sequence	ND4			
2726	Coding sequence	ND4			
2918	Coding sequence	ND4			
3110	Coding sequence	ND4			
3119	Coding sequence	ND4			
3233	Coding sequence	ND4			
3272	Coding sequence	ND4			
3356	Coding sequence	ND4			
3360	Coding sequence	ND4			
3383	Coding sequence	ND4			
3518	Coding sequence	ND5			
3563	Coding sequence	ND5			
3652	Coding sequence	ND5			
3653	Coding sequence	ND5			
3752	Coding sequence	ND5			
3875	Coding sequence	ND5			
4001	Coding sequence	ND5			
4138	Coding sequence	ND5			
4507	Coding sequence	ND5			
4573	Coding sequence	ND5			
4585	Coding sequence	ND5			
4747	Coding sequence	ND5			
4759	Coding sequence	ND5			
4915	Coding sequence	ND5			
4925	Coding sequence	ND5			

Site	Region	Gene	Gene	Number of SNPs	Proportion of coding-region SNPs (%)
4952	Coding sequence	ND5			
4957	Coding sequence	ND5			
5151	Non-coding region				
5165	Non-coding region				
5243	Non-coding region				
5472	Coding sequence	ND3			
5810	Coding sequence	ND3			
5938	Coding sequence	COX3			
6129	Coding sequence	COX3			
6151	Coding sequence	COX3			
6286	Coding sequence	COX3			
6298	Coding sequence	COX3			
6543	Coding sequence	COX3			
6754	Coding sequence	ATP6			
7023	Coding sequence	ATP6			
7044	Coding sequence	ATP6			
7188	Coding sequence	ATP6			
7259	Coding sequence	ATP6			
7260	Coding sequence	ATP6			
7262	Coding sequence	ATP6			
7281	Coding sequence	ATP6			
7311	Coding sequence	ATP6			
7359	Coding sequence	ATP8			
7393	Coding sequence	ATP8			
7409	Coding sequence	ATP8			
7414	Coding sequence	ATP8			
7533	Non-coding region				

Site	Region	Gene	Gene	Number of SNPs	Proportion of coding-region SNPs (%)
7547	Non-coding region				
7635	Coding sequence	COX2			
7863	Coding sequence	COX2			
7923	Coding sequence	COX2			
7971	Coding sequence	COX2			
8034	Coding sequence	COX2			
8088	Coding sequence	COX2			
8201	Coding sequence	COX2			
8228	Coding sequence	COX2			
8404	Coding sequence	COX1			
8498	Coding sequence	COX1			
8509	Coding sequence	COX1			
8536	Coding sequence	COX1			
8566	Coding sequence	COX1			
8641	Coding sequence	COX1			
9051	Coding sequence	COX1			
9078	Coding sequence	COX1			
9129	Coding sequence	COX1			
9211	Coding sequence	COX1			
9214	Coding sequence	COX1			
9577	Coding sequence	COX1			
9748	Coding sequence	COX1			
9771	Coding sequence	COX1			
9810	Coding sequence	COX1			
9846	Coding sequence	COX1			
10015	Non-coding region				
10062	Non-coding region				
10123	Coding sequence	ND2			

Site	Region	Gene	Gene	Number of SNPs	Proportion of coding-region SNPs (%)
10424	Coding sequence	ND2			
10480	Coding sequence	ND2			
10520	Coding sequence	ND2			
10525	Coding sequence	ND2			
10614	Coding sequence	ND2			
10615	Coding sequence	ND2			
10677	Coding sequence	ND2			
10748	Coding sequence	ND2			
10896	Coding sequence	ND2			
10974	Coding sequence	ND2			
11501	Non-coding region				
11503	Non-coding region				
11505	Non-coding region				
11506	Non-coding region				
11510	Non-coding region				
11511	Non-coding region				
11512	Non-coding region				
11515	Non-coding region				
11521	Non-coding region				
11522	Non-coding region				
11524	Non-coding region				
11526	Non-coding region				
11538	Non-coding region				

Site	Region	Gene	Gene	Number of SNPs	Proportion of coding-region SNPs (%)
11539	Non-coding region				
11540	Non-coding region				
11557	Non-coding region				
11563	Non-coding region				
11575	Non-coding region				
11579	Non-coding region				
11580	Non-coding region				
11582	Non-coding region				
11639	Non-coding region				
11690	Non-coding region				
11702	Non-coding region				
11703	Non-coding region				
11708	Non-coding region				
11710	Non-coding region				
13159	Non-coding region				
13163	Non-coding region				
13166	Non-coding region				
13167	Non-coding region				
13168	Non-coding region				
13281	Non-coding region				

Site	Region	Gene	Gene	Number of SNPs	Proportion of coding-region SNPs (%)
13288	Non-coding region				
13296	Non-coding region				
13297	Non-coding region				
13311	Non-coding region				
13322	Non-coding region				
13497	Non-coding region				
13694	Non-coding region				
13843	Non-coding region				
14198	Non-coding region				
14237	Non-coding region				
14251	Non-coding region				
14381	Non-coding region				
14454	Non-coding region				
14495	Non-coding region				
14503	Non-coding region				
14579	Non-coding region				
14835	Non-coding region				
15005	Non-coding region				
15008	Non-coding region				
15133	Non-coding region				

Site	Region	Gene	Gene	Number of SNPs	Proportion of coding-region SNPs (%)
15544	Coding sequence	ND1			
15608	Coding sequence	ND1			
15638	Coding sequence	ND1			
15693	Coding sequence	ND1			
15719	Coding sequence	ND1			
15775	Coding sequence	ND1			
15872	Coding sequence	ND1			
15934	Coding sequence	ND1			
15946	Coding sequence	ND1			
15994	Coding sequence	ND1			
16087	Coding sequence	ND1			

Table S5. Thirty-two fixed nucleotide differences between the Asian and American *Diaphorina citri* mitochondrial lineages (132-genome dataset excluding the reference sequence MF426268.1): genomic positions (referenced to the MF426268.1 mitochondrial genome backbone, 15,400 bp), gene annotations and functional classification.

**Gene**	**Position (MF426268.1)**	**Strand**	**Asian allele**	**American allele**	**Type**	**AA change**
Control region	20		G	A	non-coding	-
CYTB	894	-	T	C	Syn	G->G
ND6	1480	-	G	A	NonSyn	L->F
ND4	2696	+	C	T	Syn	H->H
ND4	3233	+	T	C	Syn	F->F
ND4	3272	+	A	G	Syn	M->M
ND5	3653	+	G	A	NonSyn	V->M
ND5	4001	+	C	T	NonSyn	L->F
ND5	4585	+	A	G	Syn	G->G
ND5	4747	+	G	A	Syn	V->V
ND5	4915	+	A	G	Syn	K->K
trnF	5151		T	C	non-coding	-
trnS	5241		T	C	non-coding	-
ND3	5468	-	A	G	Syn	N->N
COX3	6147	-	T	C	Syn	S->S
ATP6	7256	-	C	T	Syn	S->S
ATP6	7277	-	C	T	Syn	W->W
ATP8	7389	-	A	C	NonSyn	Y->D
ATP8	7405	-	G	A	Syn	I->I
COX2	8197	-	T	A	NonSyn	M->L
COX1	8532	-	C	T	Syn	W->W
COX1	9047	-	T	C	NonSyn	T->A
COX1	9573	-	C	T	Syn	W->W
tRNA cluster	10011		G	T	non-coding	-
rrn12	12881		G	A	non-coding	-

rrn16	13438		C	T	non-coding	-
rrn16	13641		C	T	non-coding	-
rrn16	13682		G	A	non-coding	-
rrn16	13766		A	G	non-coding	-
ND1	14793	+	G	T	NonSyn	A->S
ND1	14960	+	T	C	Syn	I->I
Control region	15179		A	G	non-coding	-

Table S6. Population-specific (private) polymorphic sites and allele frequencies in the Asian and American *D. citri* mitochondrial lineages.

**American**				**Asian**				**Region**
Site#	States	State frequencies		Site#	States	State frequencies		
1	1	A : 1.0000		1	1	G : 1.0000		Non-coding
2	1	T : 1.0000		2	2	T : 0.9890	C : 0.0110	Non-coding
3	2	G : 0.9762	A : 0.0238	3	1	A : 1.0000		Non-coding
4	1	G : 1.0000		4	2	G : 0.9890	A : 0.0110	CYTB
5	1	G : 1.0000		5	2	G : 0.9121	T : 0.0879	CYTB
6	1	G : 1.0000		6	2	G : 0.9890	A : 0.0110	CYTB
7	1	A : 1.0000		7	2	A : 0.9890	G : 0.0110	CYTB
8	1	A : 1.0000		8	2	C : 0.9670	T : 0.0330	CYTB
9	1	A : 1.0000		9	2	A : 0.9780	G : 0.0220	CYTB
10	1	T : 1.0000		10	2	T : 0.9890	C : 0.0110	CYTB
11	1	C : 1.0000		11	2	C : 0.9890	T : 0.0110	CYTB
12	1	C : 1.0000		12	2	C : 0.9890	T : 0.0110	CYTB
13	1	A : 1.0000		13	2	A : 0.9670	G : 0.0330	CYTB
14	1	C : 1.0000		14	1	T : 1.0000		CYTB
15	1	C : 1.0000		15	2	C : 0.9890	T : 0.0110	CYTB
16	1	T : 1.0000		16	2	T : 0.9890	C : 0.0110	CYTB
17	1	T : 1.0000		17	2	T : 0.9890	C : 0.0110	CYTB
18	1	C : 1.0000		18	2	C : 0.9780	T : 0.0220	CYTB
19	1	C : 1.0000		19	2	C : 0.9890	T : 0.0110	CYTB
20	1	A : 1.0000		20	2	A : 0.9670	G : 0.0330	ND6
21	1	A : 1.0000		21	1	G : 1.0000		ND6
22	1	G : 1.0000		22	2	G : 0.9890	C : 0.0110	ND6
23	1	G : 1.0000		23	2	G : 0.9890	T : 0.0110	ND6
24	1	C : 1.0000		24	2	C : 0.9890	T : 0.0110	ND6
25	2	G : 0.9762	A : 0.0238	25	1	A : 1.0000		ND6
26	1	C : 1.0000		26	2	C : 0.9890	T : 0.0110	ND6
27	1	A : 1.0000		27	2	A : 0.9890	T : 0.0110	ND6
28	1	C : 1.0000		28	2	C : 0.9890	T : 0.0110	Non-coding
29	1	A : 1.0000		29	2	A : 0.9890	G : 0.0110	ND4L
30	1	T : 1.0000		30	2	T : 0.9890	C : 0.0110	ND4
31	2	C : 0.9762	T : 0.0238	31	1	T : 1.0000		ND4
32	1	T : 1.0000		32	1	C : 1.0000		ND4
33	1	A : 1.0000		33	2	A : 0.9890	T : 0.0110	ND4
34	1	G : 1.0000		34	2	G : 0.9890	T : 0.0110	ND4
35	2	C : 0.9762	T : 0.0238	35	1	T : 1.0000		ND4
36	1	C : 1.0000		36	2	C : 0.9890	T : 0.0110	ND4
37	1	C : 1.0000		37	1	T : 1.0000		ND4
38	1	G : 1.0000		38	1	A : 1.0000		ND4
39	1	C : 1.0000		39	2	C : 0.9670	T : 0.0330	ND4
40	1	T : 1.0000		40	2	T : 0.9780	C : 0.0220	ND4
41	1	C : 1.0000		41	2	T : 0.9890	C : 0.0110	ND4

American				Asian				Region
42	1	C : 1.0000		42	2	C : 0.9011	T : 0.0989	ND5
43	1	A : 1.0000		43	2	A : 0.9780	G : 0.0220	ND5
44	1	A : 1.0000		44	2	A : 0.9890	G : 0.0110	ND5
45	1	A : 1.0000		45	1	G : 1.0000		ND5
46	1	C : 1.0000		46	2	C : 0.9890	T : 0.0110	ND5
47	1	A : 1.0000		47	2	A : 0.9890	G : 0.0110	ND5
48	1	T : 1.0000		48	1	C : 1.0000		ND5
49	1	T : 1.0000		49	2	T : 0.9890	C : 0.0110	ND5
50	1	C : 1.0000		50	2	C : 0.9890	T : 0.0110	ND5
51	1	T : 1.0000		51	2	T : 0.9890	C : 0.0110	ND5
52	1	G : 1.0000		52	1	A : 1.0000		ND5
53	1	A : 1.0000		53	1	G : 1.0000		ND5
54	2	A : 0.9762	G : 0.0238	54	1	G : 1.0000		ND5
55	1	G : 1.0000		55	1	A : 1.0000		ND5
56	1	C : 1.0000		56	2	C : 0.9890	T : 0.0110	ND5
57	1	A : 1.0000		57	2	A : 0.9890	G : 0.0110	ND5
58	1	A : 1.0000		58	2	A : 0.9890	G : 0.0110	ND5
59	1	C : 1.0000		59	1	T : 1.0000		Non-coding
60	2	G : 0.9762	A : 0.0238	60	1	A : 1.0000		Non-coding
61	1	C : 1.0000		61	1	T : 1.0000		Non-coding
62	1	G : 1.0000		62	1	A : 1.0000		Non-coding
63	1	A : 1.0000		63	2	G : 0.9011	A : 0.0989	Non-coding
64	1	T : 1.0000		64	2	T : 0.9890	C : 0.0110	COX3
65	1	T : 1.0000		65	2	T : 0.9890	A : 0.0110	COX3
66	1	C : 1.0000		66	1	T : 1.0000		COX3
67	1	A : 1.0000		67	2	A : 0.9890	G : 0.0110	COX3
68	1	G : 1.0000		68	2	G : 0.9890	A : 0.0110	COX3
69	1	T : 1.0000		69	2	T : 0.9890	A : 0.0110	COX3
70	1	A : 1.0000		70	2	A : 0.9890	G : 0.0110	ATP6
71	1	T : 1.0000		71	2	T : 0.9560	C : 0.0440	ATP6
72	1	T : 1.0000		72	2	T : 0.9780	C : 0.0220	ATP6
73	1	T : 1.0000		73	2	T : 0.9890	C : 0.0110	ATP6
74	1	T : 1.0000		74	2	T : 0.9890	C : 0.0110	ATP6
75	1	T : 1.0000		75	1	C : 1.0000		ATP6
76	1	A : 1.0000		76	2	A : 0.9560	C : 0.0440	ATP6
77	1	T : 1.0000		77	1	C : 1.0000		ATP6
78	1	A : 1.0000		78	2	A : 0.9890	G : 0.0110	ATP6
79	1	G : 1.0000		79	2	G : 0.9890	T : 0.0110	ATP8
80	1	C : 1.0000		80	1	A : 1.0000		ATP8
81	1	A : 1.0000		81	1	G : 1.0000		ATP8
82	1	T : 1.0000		82	2	T : 0.9890	A : 0.0110	ATP8
83	1	T : 1.0000		83	2	T : 0.9780	C : 0.0220	Non-coding
84	1	T : 1.0000		84	2	T : 0.9670	C : 0.0330	Non-coding
85	1	T : 1.0000		85	2	T : 0.9890	C : 0.0110	COX2

American				Asian				Region
86	1	T : 1.0000		86	2	T : 0.9780	C : 0.0220	COX2
87	1	G : 1.0000		87	2	G : 0.9890	A : 0.0110	COX2
88	1	A : 1.0000		88	2	G : 0.9121	A : 0.0879	COX2
89	1	C : 1.0000		89	2	C : 0.9780	T : 0.0220	COX2
90	2	A : 0.9762	G : 0.0238	90	2	G : 0.9890	A : 0.0110	COX2
91	1	A : 1.0000		91	1	T : 1.0000		COX2
92	1	A : 1.0000		92	2	A : 0.9890	G : 0.0110	COX2
93	1	C : 1.0000		93	2	C : 0.9780	T : 0.0220	COX1
94	1	A : 1.0000		94	2	A : 0.9890	G : 0.0110	COX1
95	1	T : 1.0000		95	2	T : 0.9780	A : 0.0220	COX1
96	1	T : 1.0000		96	1	C : 1.0000		COX1
97	1	G : 1.0000		97	2	G : 0.9890	A : 0.0110	COX1
98	1	T : 1.0000		98	2	T : 0.9890	A : 0.0110	COX1
99	1	C : 1.0000		99	1	T : 1.0000		COX1
100	1	T : 1.0000		100	2	T : 0.9890	C : 0.0110	COX1
101	1	A : 1.0000		101	2	G : 0.9890	A : 0.0110	COX1
102	1	A : 1.0000		102	2	A : 0.9890	T : 0.0110	COX1
103	1	T : 1.0000		103	2	T : 0.9890	C : 0.0110	COX1
104	1	T : 1.0000		104	1	C : 1.0000		COX1
105	1	T : 1.0000		105	2	C : 0.9780	T : 0.0220	COX1
106	1	A : 1.0000		106	2	A : 0.9890	T : 0.0110	COX1
107	1	C : 1.0000		107	2	C : 0.9780	T : 0.0220	COX1
108	1	T : 1.0000		108	2	T : 0.9890	G : 0.0110	COX1
109	1	T : 1.0000		109	1	G : 1.0000		Non-coding
110	1	T : 1.0000		110	2	T : 0.9890	C : 0.0110	Non-coding
111	2	A : 0.9762	G : 0.0238	111	1	G : 1.0000		Non-coding
112	2	C : 0.9762	T : 0.0238	112	1	T : 1.0000		Non-coding
113	1	G : 1.0000		113	2	G : 0.9890	A : 0.0110	ND2
114	1	T : 1.0000		114	2	T : 0.9890	G : 0.0110	ND2
115	1	G : 1.0000		115	2	G : 0.9890	A : 0.0110	ND2
116	1	G : 1.0000		116	2	G : 0.9890	A : 0.0110	ND2
117	1	A : 1.0000		117	2	A : 0.9890	G : 0.0110	ND2
118	1	G : 1.0000		118	2	G : 0.9890	A : 0.0110	ND2
119	1	G : 1.0000		119	2	G : 0.9890	A : 0.0110	ND2
120	1	T : 1.0000		120	2	T : 0.9890	C : 0.0110	ND2
121	1	G : 1.0000		121	2	G : 0.9890	A : 0.0110	ND2
122	2	A : 0.9762	G : 0.0238	122	1	A : 1.0000		ND2
123	2	T : 0.9762	C : 0.0238	123	1	T : 1.0000		Non-coding
124	2	A : 0.9762	C : 0.0238	124	1	A : 1.0000		Non-coding
125	2	G : 0.9762	C : 0.0238	125	1	G : 1.0000		Non-coding
126	2	A : 0.9762	G : 0.0238	126	1	A : 1.0000		Non-coding
127	2	T : 0.9762	A : 0.0238	127	1	T : 1.0000		Non-coding
128	2	T : 0.9762	C : 0.0238	128	1	T : 1.0000		Non-coding
129	2	T : 0.9762	A : 0.0238	129	1	T : 1.0000		Non-coding

American				Asian				Region
130	2	T : 0.9762	C : 0.0238	130	1	T : 1.0000		Non-coding
131	2	A : 0.9762	T : 0.0238	131	1	A : 1.0000		Non-coding
132	2	T : 0.9762	C : 0.0238	132	1	T : 1.0000		Non-coding
133	2	A : 0.9762	G : 0.0238	133	1	A : 1.0000		Non-coding
134	2	A : 0.9762	C : 0.0238	134	2	A : 0.9670	T : 0.0330	Non-coding
135	2	T : 0.3095	A : 0.6905	135	2	A : 0.8681	T : 0.1319	Non-coding
136	2	C : 0.2857	T : 0.7143	136	3	T : 0.8352	A : 0.0330 ; C : 0.1319	Non-coding
137	2	T : 0.9762	C : 0.0238	137	1	T : 1.0000		Non-coding
138	2	T : 0.9762	C : 0.0238	138	1	T : 1.0000		Non-coding
139	2	A : 0.9762	G : 0.0238	139	1	A : 1.0000		Non-coding
140	2	T : 0.9762	C : 0.0238	140	1	T : 1.0000		Non-coding
141	2	T : 0.9762	C : 0.0238	141	1	T : 1.0000		Non-coding
142	2	A : 0.9762	G : 0.0238	142	1	A : 1.0000		Non-coding
143	2	A : 0.9762	T : 0.0238	143	1	A : 1.0000		Non-coding
144	2	A : 0.9762	T : 0.0238	144	1	A : 1.0000		Non-coding
145	2	A : 0.9762	T : 0.0238	145	1	A : 1.0000		Non-coding
146	2	A : 0.9762	T : 0.0238	146	1	A : 1.0000		Non-coding
147	2	A : 0.9762	C : 0.0238	147	1	A : 1.0000		Non-coding
148	2	T : 0.9762	G : 0.0238	148	1	T : 1.0000		Non-coding
149	1	T : 1.0000		149	2	T : 0.9890	G : 0.0110	Non-coding
150	1	A : 1.0000		150	2	A : 0.9890	C : 0.0110	Non-coding
151	1	T : 1.0000		151	2	T : 0.9890	G : 0.0110	Non-coding
152	1	A : 1.0000		152	2	A : 0.9890	T : 0.0110	Non-coding
153	1	G : 1.0000		153	2	G : 0.9890	A : 0.0110	Non-coding
154	2	A : 0.9762	G : 0.0238	154	1	A : 1.0000		Non-coding
155	2	A : 0.9762	G : 0.0238	155	1	A : 1.0000		Non-coding
156	2	T : 0.9762	A : 0.0238	156	1	T : 1.0000		Non-coding
157	1	C : 1.0000		157	2	C : 0.9780	T : 0.0220	Non-coding
158	1	G : 1.0000		158	2	G : 0.9890	A : 0.0110	Non-coding
159	2	T : 0.9762	A : 0.0238	159	1	T : 1.0000		Non-coding
160	1	A : 1.0000		160	2	A : 0.9890	G : 0.0110	Non-coding
161	1	A : 1.0000		161	1	G : 1.0000		Non-coding
162	2	A : 0.0952	G : 0.9048	162	1	A : 1.0000		Non-coding
163	1	G : 1.0000		163	2	G : 0.9890	A : 0.0110	Non-coding
164	1	A : 1.0000		164	2	G : 0.9011	A : 0.0989	Non-coding
165	1	T : 1.0000		165	1	C : 1.0000		Non-coding
166	1	T : 1.0000		166	2	T : 0.6044	C : 0.3956	Non-coding
167	1	T : 1.0000		167	1	C : 1.0000		Non-coding
168	1	A : 1.0000		168	1	G : 1.0000		Non-coding
169	1	C : 1.0000		169	2	C : 0.9890	T : 0.0110	Non-coding
170	1	G : 1.0000		170	1	A : 1.0000		Non-coding
171	1	G : 1.0000		171	2	G : 0.9890	A : 0.0110	Non-coding
172	1	A : 1.0000		172	2	A : 0.9890	G : 0.0110	Non-coding
173	1	G : 1.0000		173	2	G : 0.9890	A : 0.0110	Non-coding

American			Asian				Region
174	1	C : 1.0000	174	2	C : 0.9890	T : 0.0110	Non-coding
175	1	T : 1.0000	175	2	T : 0.9890	C : 0.0110	ND1
176	1	T : 1.0000	176	1	G : 1.0000		ND1
177	1	G : 1.0000	177	2	G : 0.9890	A : 0.0110	ND1
178	1	T : 1.0000	178	2	T : 0.9780	A : 0.0220	ND1
179	1	A : 1.0000	179	2	A : 0.9670	G : 0.0330	ND1
180	1	C : 1.0000	180	1	T : 1.0000		ND1
181	1	C : 1.0000	181	2	C : 0.9890	T : 0.0110	ND1
182	1	A : 1.0000	182	2	A : 0.9890	G : 0.0110	ND1
183	1	T : 1.0000	183	2	T : 0.9890	C : 0.0110	ND1
184	1	G : 1.0000	184	1	A : 1.0000		ND1
185	1	G : 1.0000	185	2	G : 0.9780	A : 0.0220	ND1

Table S7. Nucleotide variants and allele frequency differences between the Yunnan-Guizhou Plateau and lowland plains populations of *D. citri*.

**Plateau**				**Plains**				**Region**
Site#	States	State frequencies		Site#	States	State frequencies		
1	1	T : 1.0000		1	2	T : 0.9877	C : 0.0123	CYTB
2	1	G : 1.0000		2	2	G : 0.9877	A : 0.0123	CYTB
3	2	T : 0.8889	G : 0.1111	3	1	G : 1.0000		CYTB
4	1	G : 1.0000		4	2	G : 0.9877	A : 0.0123	CYTB
5	1	A : 1.0000		5	2	A : 0.9877	G : 0.0123	CYTB
6	1	C : 1.0000		6	2	C : 0.9630	T : 0.0370	CYTB
7	1	A : 1.0000		7	2	A : 0.9753	G : 0.0247	CYTB
8	1	T : 1.0000		8	2	T : 0.9877	C : 0.0123	CYTB
9	1	C : 1.0000		9	2	C : 0.9877	T : 0.0123	CYTB
10	1	C : 1.0000		10	2	C : 0.9877	T : 0.0123	CYTB
11	2	A : 0.6667	G : 0.3333	11	1	A : 1.0000		CYTB
12	1	C : 1.0000		12	2	C : 0.9877	T : 0.0123	CYTB
13	1	T : 1.0000		13	2	T : 0.9877	C : 0.0123	CYTB
14	2	T : 0.8889	C : 0.1111	14	1	T : 1.0000		CYTB
15	1	C : 1.0000		15	2	C : 0.9753	T : 0.0247	CYTB
16	1	C : 1.0000		16	2	C : 0.9877	T : 0.0123	CYTB
17	1	A : 1.0000		17	2	A : 0.9630	G : 0.0370	ND6
18	1	G : 1.0000		18	2	G : 0.9877	C : 0.0123	ND6
19	1	G : 1.0000		19	2	G : 0.9877	T : 0.0123	ND6
20	1	C : 1.0000		20	2	C : 0.9877	T : 0.0123	ND6
21	1	C : 1.0000		21	2	C : 0.9877	T : 0.0123	ND6
22	1	A : 1.0000		22	2	A : 0.9877	T : 0.0123	ND6
23	1	C : 1.0000		23	2	C : 0.9877	T : 0.0123	Non-coding
24	1	A : 1.0000		24	2	A : 0.9877	G : 0.0123	ND4L
25	1	T : 1.0000		25	2	T : 0.9877	C : 0.0123	ND4
26	1	A : 1.0000		26	2	A : 0.9877	T : 0.0123	ND4
27	1	G : 1.0000		27	2	G : 0.9877	T : 0.0123	ND4
28	2	C : 0.8889	T : 0.1111	28	1	C : 1.0000		ND4
29	2	C : 0.8889	T : 0.1111	29	2	C : 0.9753	T : 0.0247	ND4
30	1	T : 1.0000		30	2	T : 0.9753	C : 0.0247	ND4
31	1	T : 1.0000		31	2	T : 0.9877	C : 0.0123	ND4
32	1	C : 1.0000		32	2	C : 0.8889	T : 0.1111	ND5
33	1	A : 1.0000		33	2	A : 0.9753	G : 0.0247	ND5
34	1	C : 1.0000		34	2	C : 0.9877	T : 0.0123	ND5
35	1	A : 1.0000		35	2	A : 0.9877	G : 0.0123	ND5
36	2	C : 0.1111	T : 0.8889	36	1	T : 1.0000		ND5
37	1	C : 1.0000		37	2	C : 0.9877	T : 0.0123	ND5
38	1	T : 1.0000		38	2	T : 0.9877	C : 0.0123	ND5
39	2	C : 0.8889	T : 0.1111	39	1	C : 1.0000		ND5
40	1	A : 1.0000		40	2	A : 0.9877	G : 0.0123	ND5
41	2	A : 0.8889	G : 0.1111	41	1	A : 1.0000		ND5

Plateau				Plains				Region
42	1	A : 1.0000		42	1	G : 1.0000		trnG
43	1	T : 1.0000		43	2	T : 0.9877	C : 0.0123	COX3
44	1	T : 1.0000		44	2	T : 0.9877	A : 0.0123	COX3
45	1	A : 1.0000		45	2	A : 0.9877	G : 0.0123	COX3
46	1	G : 1.0000		46	2	G : 0.9877	A : 0.0123	COX3
47	1	T : 1.0000		47	2	T : 0.9877	A : 0.0123	COX3
48	1	A : 1.0000		48	2	A : 0.9877	G : 0.0123	ATP6
49	1	T : 1.0000		49	2	T : 0.9506	C : 0.0494	ATP6
50	1	T : 1.0000		50	2	T : 0.9753	C : 0.0247	ATP6
51	1	T : 1.0000		51	2	T : 0.9877	C : 0.0123	ATP6
52	1	T : 1.0000		52	2	T : 0.9877	C : 0.0123	ATP6
53	1	A : 1.0000		53	2	A : 0.9506	C : 0.0494	ATP6
54	1	A : 1.0000		54	2	A : 0.9877	G : 0.0123	ATP6
55	1	G : 1.0000		55	2	G : 0.9877	T : 0.0123	ATP8
56	1	T : 1.0000		56	2	T : 0.9877	A : 0.0123	ATP8
57	2	T : 0.7778	C : 0.2222	57	1	T : 1.0000		trnD
58	1	T : 1.0000		58	2	T : 0.9630	C : 0.0370	trnD
59	2	T : 0.8889	C : 0.1111	59	1	T : 1.0000		COX2
60	1	T : 1.0000		60	2	T : 0.9753	C : 0.0247	COX2
61	1	G : 1.0000		61	2	G : 0.9877	A : 0.0123	COX2
62	2	A : 0.8889	G : 0.1111	62	1	G : 1.0000		COX2
63	1	C : 1.0000		63	2	C : 0.9753	T : 0.0247	COX2
64	1	G : 1.0000		64	2	G : 0.9877	A : 0.0123	COX2
65	1	A : 1.0000		65	2	A : 0.9877	G : 0.0123	COX2
66	1	C : 1.0000		66	2	C : 0.9753	T : 0.0247	COX1
67	1	A : 1.0000		67	2	A : 0.9877	G : 0.0123	COX1
68	1	T : 1.0000		68	2	T : 0.9753	A : 0.0247	COX1
69	1	G : 1.0000		69	2	G : 0.9877	A : 0.0123	COX1
70	2	T : 0.8889	A : 0.1111	70	1	T : 1.0000		COX1
71	1	T : 1.0000		71	2	T : 0.9877	C : 0.0123	COX1
72	1	G : 1.0000		72	2	G : 0.9877	A : 0.0123	COX1
73	1	A : 1.0000		73	2	A : 0.9877	T : 0.0123	COX1
74	1	T : 1.0000		74	2	T : 0.9877	C : 0.0123	COX1
75	1	C : 1.0000		75	2	C : 0.9753	T : 0.0247	COX1
76	2	A : 0.8889	T : 0.1111	76	1	A : 1.0000		COX1
77	1	C : 1.0000		77	2	C : 0.9753	T : 0.0247	COX1
78	1	T : 1.0000		78	2	T : 0.9877	G : 0.0123	COX1
79	2	T : 0.8889	C : 0.1111	79	1	T : 1.0000		trnW
80	1	G : 1.0000		80	2	G : 0.9877	A : 0.0123	ND2
81	1	T : 1.0000		81	2	T : 0.9877	G : 0.0123	ND2
82	1	G : 1.0000		82	2	G : 0.9877	A : 0.0123	ND2
83	1	G : 1.0000		83	2	G : 0.9877	A : 0.0123	ND2
84	1	A : 1.0000		84	2	A : 0.9877	G : 0.0123	ND2
85	1	G : 1.0000		85	2	G : 0.9877	A : 0.0123	ND2

Plateau				Plains				Region
86	1	G : 1.0000		86	2	G : 0.9877	A : 0.0123	ND2
87	1	T : 1.0000		87	2	T : 0.9877	C : 0.0123	ND2
88	1	G : 1.0000		88	2	G : 0.9877	A : 0.0123	ND2
89	1	T : 1.0000		89	2	T : 0.9877	A : 0.0123	Non-coding
90	1	T : 1.0000		90	2	T : 0.9877	G : 0.0123	Non-coding
91	1	T : 1.0000		91	2	T : 0.7531	A : 0.2469	Non-coding
92	1	A : 1.0000		92	2	A : 0.7531	T : 0.2469	Non-coding
93	1	T : 1.0000		93	2	T : 0.7531	C : 0.2469	Non-coding
94	1	C : 1.0000		94	2	C : 0.7531	A : 0.2469	Non-coding
95	1	C : 1.0000		95	2	C : 0.9877	T : 0.0123	Non-coding
96	1	A : 1.0000		96	2	A : 0.9877	G : 0.0123	Non-coding
97	1	G : 1.0000		97	2	G : 0.9877	A : 0.0123	Non-coding
98	1	G : 1.0000		98	2	G : 0.9877	A : 0.0123	Non-coding
99	1	C : 1.0000		99	2	C : 0.9877	A : 0.0123	Non-coding
100	2	G : 0.4444	A : 0.5556	100	2	A : 0.8395	G : 0.1605	Non-coding
101	1	T : 1.0000		101	2	T : 0.9877	G : 0.0123	Non-coding
102	1	A : 1.0000		102	2	A : 0.9877	C : 0.0123	Non-coding
103	1	T : 1.0000		103	2	T : 0.9877	G : 0.0123	Non-coding
104	1	A : 1.0000		104	2	A : 0.9877	T : 0.0123	Non-coding
105	1	G : 1.0000		105	2	G : 0.9877	A : 0.0123	Non-coding
106	1	C : 1.0000		106	2	C : 0.9753	T : 0.0247	Non-coding
107	1	G : 1.0000		107	2	G : 0.9877	A : 0.0123	Non-coding
108	1	A : 1.0000		108	2	A : 0.9877	G : 0.0123	rrn12
109	1	G : 1.0000		109	2	G : 0.9877	A : 0.0123	rrn12
110	1	A : 1.0000		110	1	G : 1.0000		rrn16
111	1	T : 1.0000		111	2	T : 0.5679	C : 0.4321	rrn16
112	1	C : 1.0000		112	2	C : 0.9877	T : 0.0123	rrn16
113	1	G : 1.0000		113	2	G : 0.9877	A : 0.0123	rrn16
114	1	A : 1.0000		114	2	A : 0.9877	G : 0.0123	rrn16
115	1	G : 1.0000		115	2	G : 0.9877	A : 0.0123	rrn16
116	1	C : 1.0000		116	2	C : 0.9877	T : 0.0123	rrn16
117	1	T : 1.0000		117	2	T : 0.9877	C : 0.0123	ND1
118	1	G : 1.0000		118	2	G : 0.9877	A : 0.0123	ND1
119	2	T : 0.7778	A : 0.2222	119	1	T : 1.0000		ND1
120	1	A : 1.0000		120	2	A : 0.9630	G : 0.0370	ND1
121	1	C : 1.0000		121	2	C : 0.9877	T : 0.0123	ND1
122	1	A : 1.0000		122	2	A : 0.9877	G : 0.0123	ND1
123	1	T : 1.0000		123	2	T : 0.9877	C : 0.0123	ND1
124	2	G : 0.7778	A : 0.2222	124	1	G : 1.0000		ND1

Table S8. Three competing demographic scenarios tested by DIYABC v2.0 for inferring divergence history among Southeast Asia, Southeast China, and Yunnan-Guizhou Plateau populations of *D. citri*.

Scenario	Demographic Model	Biological Interpretation	Graphical Representation
1	SA → SC → GY (sequential divergence)	Southeast Asia as the ancestral source, giving rise first to Southeast China, which subsequently diverged into the Yunnan-Guizhou Plateau lineage.	
2	SC as ancestral source diverging into SA and GY	Southeast China represents the oldest lineage, which later split into Southeast Asia and the Yunnan-Guizhou Plateau.	
3	GY as the earliest diverging lineage; SC and SA as sister groups	The Yunnan-Guizhou Plateau represents an ancient, independently evolving lineage. Southeast China and Southeast Asia share a more recent common ancestor (sister relationship).	

Table S9. Prior distributions and posterior estimates of demographic parameters under DIYABC Scenario 3, which was concordantly favored by both the direct and logistic-regression model-choice procedures. N1 was not identifiable: its posterior distribution increased monotonically towards the upper prior bound.

**Parameter**	**Minimum**	**Maximum**	**Prior / Remark**
N1(GY)	100000	10000000	Not identifiable: posterior increases monotonically towards the upper prior bound
N2(SC)	1000	100000	Posterior mode 5.07 × 10^4 (95% CI 1.69 × 10^4–9.74 × 10^4)
N3(SA)	50000	800000	Weakly identifiable (shallow, near-flat posterior)
t1	1000	50000	GY–SC divergence; posterior mode 3.52 × 10^4 (95% CI 8.17 × 10^3–4.90 × 10^4)
t2	10000	100000	SC–SA divergence; posterior mode 2.44 × 10^4 (95% CI 1.29 × 10^4–7.38 × 10^4)

Table S10. Likelihood-based model comparison for TreeMix analysis of five genetically assigned populations of *Diaphorina citri* (131 mitogenomes, 1,219 biallelic loci).

TreeMix model selection for population drift-tree analysis in D. citri				
Migration edges (m)	Log likelihood (lnL)	Likelihood improvement (ΔlnL)	Proportion of improvement (%)	Model interpretation
0	74.02	—	—	Maximum-likelihood drift tree (no migration)
1	74.07	0.05	48.3	* Largest gain; edge from (GY+NA) stem to SA (weight 0.045); covariance signal only
2	74.12	0.05	49.0	No meaningful improvement
3	74.13	0.003	2.7	No improvement (saturated)
4	74.13	0.000	0.0	No improvement (saturated)
* m = 1 gave the largest likelihood gain, but the improvement over m = 0 was minimal (ΔlnL = 0.05) and the inferred edge was unstable across sample compositions and bootstrap replicates (Figure S3).<br><br>TreeMix model selection for population drift-tree analysis in Diaphorina citri. TreeMix analysis was performed on 1,219 biallelic loci from five genetically assigned populations (SA, Southeast Asia, n = 4; SC, Southeast China, n = 67; GY, Yunnan-Guizhou				

TreeMix model selection for population drift-tree analysis in D. citri
Plateau, n = 8; NA, North America, n = 41; AF, Africa, n = 11; 131 mitogenomes in total; Changshun PP048996.1 and Leibo PP048997.1 assigned to SC by haplotype clustering). Models with m = 0–4 migration edges were compared by log-likelihood (-k 500, -global, -seed 20260822). Log-likelihood saturated beyond m = 1. Under m = 1, a single edge connected the branch ancestral to GY + NA to SA (weight = 0.045); this edge was unstable across sample compositions and bootstrap replicates and is interpreted as a covariance signal only, not as a directional dispersal route.

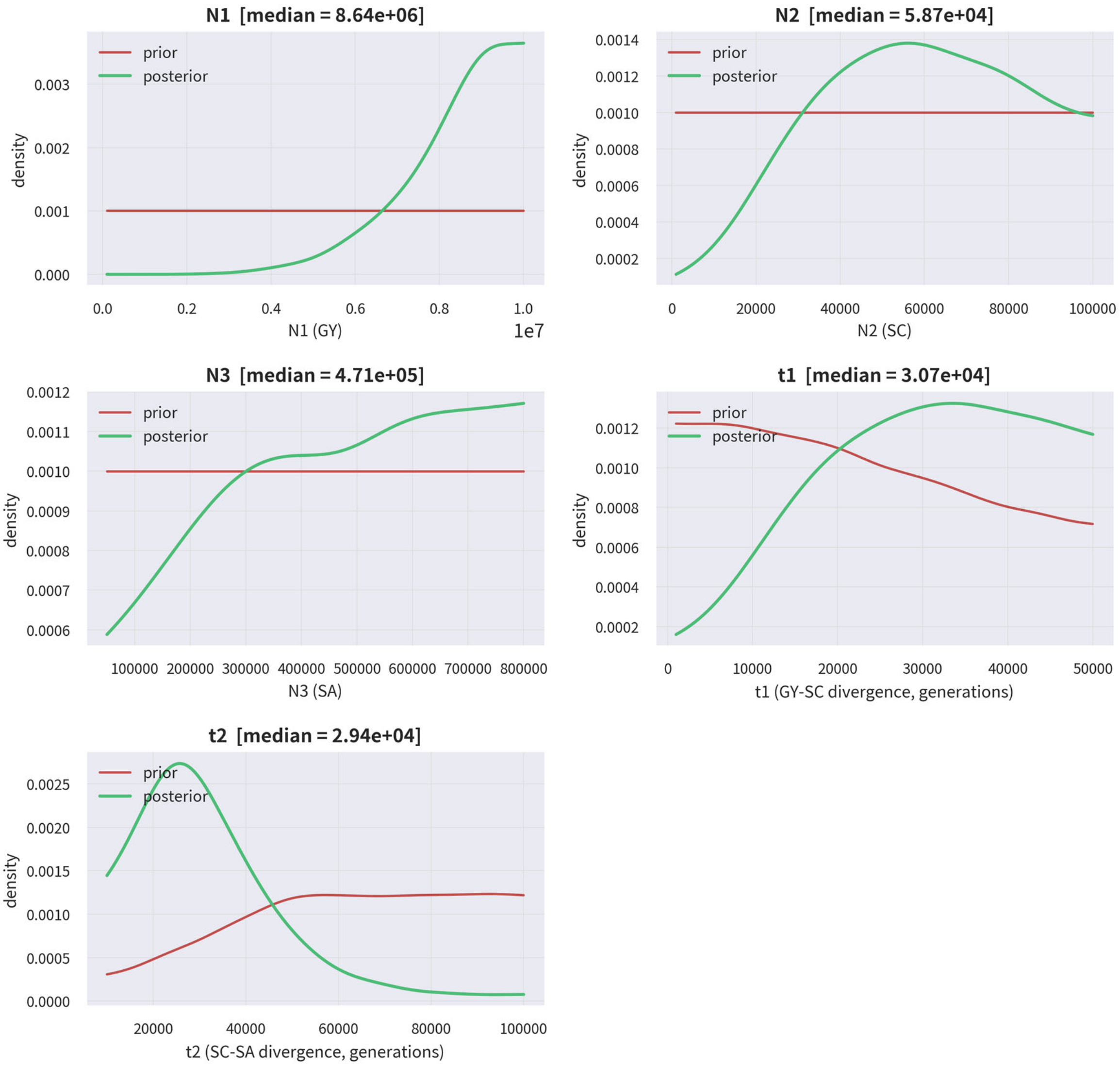

Figure S1. Prior (red) and posterior (green) distributions of demographic parameters estimated under Scenario 3 with the widened N1 prior (1×10^5–1×10^7). Values in brackets are the posterior medians: N1 = 8.64×10^6 (GY; the posterior increases monotonically towards the upper prior bound, i.e., N1 is not identifiable), N2 = 5.87×10^4 (SC), N3 = 4.71×10^5 (SA; weakly identifiable), t1 = 3.07×10^4 generations (GY–SC divergence), t2 = 2.94×10^4 generations (SC–SA divergence). Mode-based estimates with 95% intervals are given in Table S9.

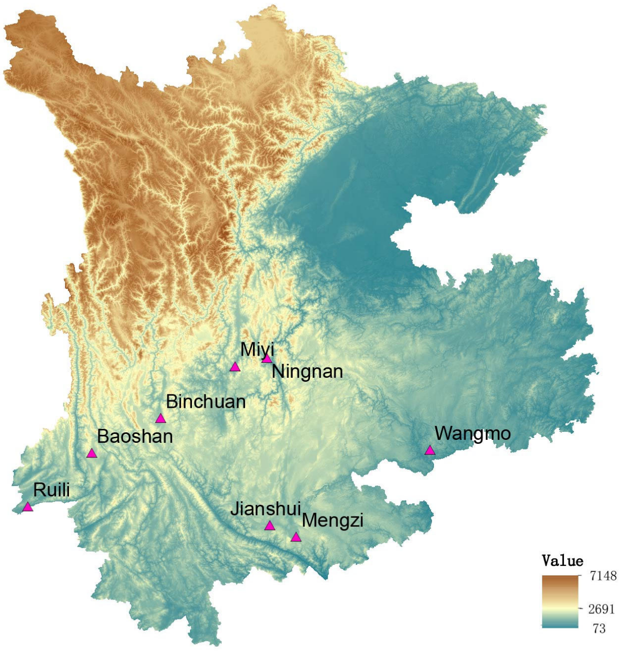

Figure S2. Geographic distribution of sampling locations in the Yunnan-Guizhou Plateau region. The map shows digital elevation model (DEM) data with hillshade effects; colors indicate elevation in metres, with warm colors indicating high elevation and cool colors indicating low elevation. Triangular symbols mark the eight sampling sites across Yunnan, Sichuan, and Guizhou provinces (elevation range 570–1,684 m, mean 1,166 m; Table S2). Scale bar and north arrow are included.

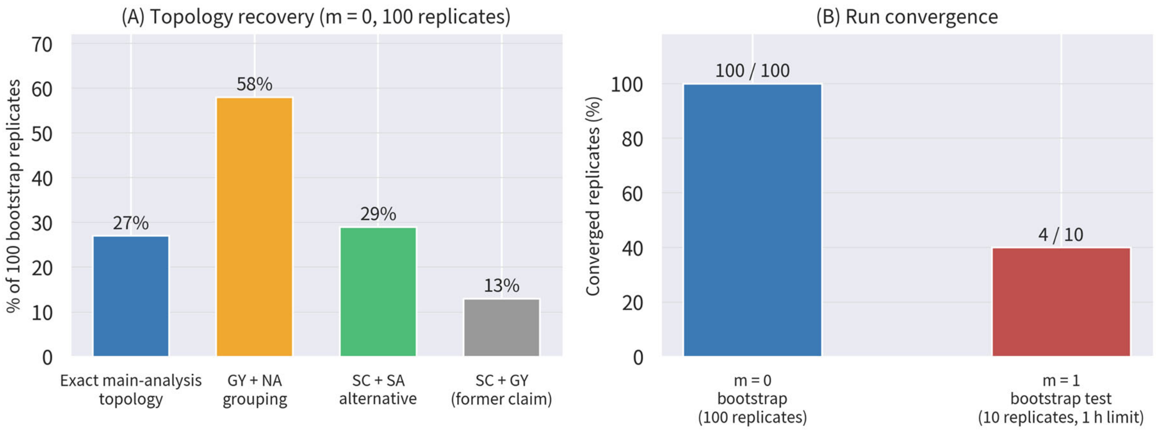

Figure S3. Bootstrap validation of the TreeMix population drift tree in *Diaphorina citri*. (A) Topology recovery across 100 bootstrap replicates under m = 0 (locus resampling with replacement; -global enabled and replicate-specific seeds): all 100 replicates converged; the exact main-analysis topology was recovered in 27% of replicates, the GY + NA grouping in 58%, the most frequent alternative grouping (SC + SA) in 29%, and the formerly claimed SC + GY sister relationship in only 13%. (B) Run convergence: 100/100 replicates converged under m = 0, whereas only 4/10 time-limited (1 h) test replicates converged under m = 1, indicating computational instability of migration-edge inference in this dataset.
